# Genetic Characteristics and Phylogenetic Relationships of 18 Anchovy Species Based on Mitochondrial Genomes in the Seas Around China

**DOI:** 10.1002/ece3.71496

**Published:** 2025-05-24

**Authors:** Wenyu Sun, Yibang Wang, Hui Zhang

**Affiliations:** ^1^ Key Laboratory of Marine Ecology and Environmental Sciences, Institute of Oceanology Chinese Academy of Sciences Qingdao China; ^2^ University of Chinese Academy of Sciences Beijing China

**Keywords:** codon usage, Engraulidae, mitochondrial genome, phylogeny

## Abstract

The anchovy family (Engraulidae) holds significant economic and ecological value in seas around China, playing a crucial role in fisheries and marine ecosystems in these regions. This study analyzed the complete mitochondrial genome data of 18 Engraulidae species from seas around China, integrating molecular evidence to systematically investigate mitochondrial genome structure, codon usage patterns, and phylogenetic relationships within the family. The mitochondrial genomes of Engraulidae exhibited a highly conserved structure, characterized by significant A + T richness and variable control region lengths. Codon usage analysis in seven *Thryssa* species revealed that base composition, particularly GC content at the third codon position (GC3s), along with purifying selection, jointly influenced codon usage patterns. Phylogenetic analyses supported the division of the 18 species into two subfamilies, Engraulinae and Coiliinae, and highlighted variability in the phylogenetic placement of *Setipinna* depending on the inclusion of third codon positions. Furthermore, the genus *Thryssa* was supported to be polyphyletic: *T. baelama* and *T. kammalensis* formed one clade, while *T. dussumieri* , *T. hamiltonii* , *T. setirostris* , *T. vitrirostris* , and *T. mystax* constituted a separate branch. These findings provide novel molecular evidence for species identification and the taxonomic classification of Engraulidae, while offering a foundation for further exploration of their evolutionary relationships and systematic taxonomy.

## Graphical abstract

In this study, we utilized the complete mitochondrial genomes of 18 Engraulidae species to analyze their basic genomic features and reconstruct the phylogenetic relationships within the family. We examined the codon usage bias in the genus *Thryssa*, explored the evolutionary relationships among the species within the genus, and we also provide some insights into the taxonomic revision of this genus.

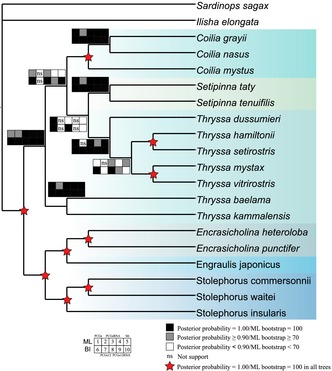

## 1 Introduction

Anchovies (Engraulidae) belong to the order Clupeiformes and the suborder Clupeoidei (Fricke 2019). They are widely distributed across the world's major oceans, with most species inhabiting tropical and subtropical regions and a few found in temperate zones (Bloom and Lovejoy 2012). Although the majority of anchovies are marine species, only a small number have been reported to live and breed in freshwater environments in South America and Southeast Asia (Weitzman and Vari 1988; Julio et al. 2022). In seas around China, there are 26 anchovy species classified into seven genera: *Engraulis*, *Coilia*, *Encrasicholina*, *Papuengraulis*, *Setipinna*, *Stolephorus*, and *Thryssa* (Chen 2015). Anchovies play a significant economic and ecological role in these regions and constitute an important part of fisheries in China (Wang et al. 2018; Lavoue et al. 2017; Tsai et al. 1998; Checkley et al. 2017). They are commonly used in aquaculture feeds, health supplements, and for human consumption (Barange et al. 2014). As a key forage fish for seabirds, marine fishes, and mammals, anchovies are drawing increasing public attention (Cury et al. 2011).

The classification of Engraulidae was first revised by Grande and Nelson based on fossil evidence, dividing Engraulidae into two subfamilies: Engraulinae and Coiliinae. Engraulinae consists of the genera *Engraulis*, *Stolephorus*, and *Encrasicholina*, along with the New World anchovies, whereas Coiliinae includes *Coilia*, *Lycothrissa*, *Papuengraulis*, *Setipinna*, *Thryssa*, and *Thrissina* (Grande and Nelson 1985). This classification framework has been widely adopted in phylogenetic studies of Engraulidae; however, morphology‐based classification methods have certain limitations. Engraulidae species exhibit only subtle morphological differences; they are small, soft‐bodied, and tend to degrade rapidly after being landed (Ma et al. 2010). According to traditional morphological classification, the main distinguishing characteristics for species within the genus *Thryssa* are the extent to which the maxilla reaches posteriorly and the number of gill rakers on the first gill arch (Chen 2015). However, due to these similarities and poor conditions after capture, accurate sample identification is often difficult (Zhang et al. 2016). Therefore, morphological traits alone may be insufficient for precise identification (Julio et al. 2022; Afrand et al. 2020). Additionally, morphological methods fail to differentiate species at early life stages (Gangan and Pavan‐Kumar 2019). This ambiguity in species identification hampers phylogenetic research and complicates the regulation and conservation of fishery resources.

In recent years, molecular biological techniques have been widely applied to resolve fish phylogenetic relationships and species identification, significantly clarifying classifications of morphologically indistinct species (Bhattacharya et al. 2016; Lavoue et al. 2010). The mitochondrial genome, due to its high copy number and universally available conserved primer sets, is easily accessible and contains sufficient phylogenetic information for inferences across broad taxonomic scales (Simon et al. 1994). It has been extensively used for species identification and phylogenetic analyses (Li et al. 2017; Dolotovskaya et al. 2017; Nelson et al. 2012; Zhao et al. 2013; Ma et al. 2012; Logue et al. 2013). However, current phylogenetic studies within the Engraulidae remain insufficient, and the taxonomy of the genus *Thryssa* remains particularly contentious (Lavoue et al. 2017). The non‐monophyletic nature and morphological similarity within *Thryssa* have caused considerable difficulties in its classification and nomenclature, with numerous misidentified species and cryptic species reported in the past (Hata et al. 2023; Hata and Lavoué 2024; Gangan and Pavan‐Kumar 2019). The genus *Thryssa* was initially placed within Engraulinae (Whitehead 1972) but was subsequently classified along with *Thrissina* into Coiliinae (Grande and Nelson 1985). Kottelat (2013) argued that *Thryssa* is not a valid name and recommended replacing it with *Thrissina*. Currently, *Thrissina* and *Thryssa* are generally regarded as synonyms. Previous studies have not supported *Thryssa* as a monophyletic group (Lavoue et al. 2017). If future studies confirm that *Thryssa* is indeed non‐monophyletic, separate generic names would be required for its distinct lineages. Before introducing any taxonomic or nomenclatural changes, it is essential to conduct more intensive taxonomic sampling to clearly define the composition of each lineage within *Thryssa*.

In this study, we analyzed complete mitochondrial genome sequences of 18 Engraulidae species belonging to five genera from waters adjacent to China, including three genomes previously sequenced by us and uploaded to the NCBI database. *Sardinops sagax* (Clupeidae) and *Ilisha elongata* (Pristigasteridae) were selected as outgroups. We reconstructed the phylogenetic relationships among these species and focused specifically on analyzing mitochondrial genome structure and codon usage bias in seven species of the genus *Thryssa*. Our aim was to clarify the phylogenetic relationships within the family Engraulidae using complete mitochondrial genomes and to provide a robust molecular foundation for revising the taxonomy and identification methods within *Thryssa*.

## 2 Materials and Methods

### 2.1 Sample Collection, Mitochondrial Genome Sequencing, and Assembly

Eighteen published complete mitochondrial genomes of anchovy species (Engraulidae) were obtained from the National Center for Biotechnology Information (NCBI) database, three of which were previously reported by our research group. The accession numbers are AP009616, AP017951, AP017952, AP017953, KF765500, KF938994, KJ710625, KM363243, KT985048, KX753639, MF668229, MG755269, MH465027, MH732976, MH732977, MK344186, MG786488, and MT764773. *Sardinops sagax* (family Clupeidae) with the accession number MW338734 and *Ilisha elongata* (family Pristigasteridae) with accession number AP009141 were used as the outgroup. Using the online software MITOS2 (available at Galaxy, https://usegalaxy.org/), the light‐strand replication origin and control region of these 18 mitochondrial genomes were re‐annotated. The mitochondrial genomes of the 18 anchovy species were then visualized using the Proksee online tool (https://proksee.ca/).

### 2.2 Bioinformatics Analysis

Nucleotide diversity and polymorphic site analysis were conducted using DnaSP v.6 (Rozas et al. 2017), which was also employed to calculate the non‐synonymous (*K*_a_) and synonymous (*K*_s_) substitution rates of the protein‐coding genes (PCGs). The K2P (Kimura 2‐Parameter) model, by balancing complexity and computational efficiency while distinguishing between transitions and transversions, has become widely adopted and recognized for measuring genetic distances in closely related species (Kimura 1980). The K2P pairwise genetic distances were computed using MEGA11 (Tamura et al. 2021). Geneious Prime and PhyloSuite v1.2.3 (Zhang et al. 2020) were used to analyze the nucleotide composition of the mitochondrial genomes, codon usage, and the relative synonymous codon usage (RSCU) values for each PCG. The AT‐skew[(A − T)/(A + T)] and GC‐skew [(G − C)/(G + C)] metrics were employed to measure differences in genomic DNA composition among genes.

### 2.3 Codon Analysis

The third base in a codon is typically the least conserved and most prone to variation. GC3s is an important parameter used to assess the frequency of guanine plus cytosine at the third position of synonymous codons, excluding Met, Trp, and stop codons. The Codon Adaptation Index (CAI) is a commonly used metric to determine whether codon usage in a gene sequence matches the high‐expression preference of the host species; its range is from 0 to 1, with higher values indicating a closer match to the host's optimal codon usage for high expression. The Codon Bias Index (CBI) measures the extent to which codon usage in the gene is biased toward certain commonly used codons; its value typically ranges from 0 to 1, and higher values suggest a stronger preference for specific codons, usually associated with higher expression levels in the host. The Frequency of Optimal Codons (FOP) quantifies the proportion of optimal codons used in the target gene, reflecting the gene's expression adaptability in the host; its value is between 0 and 1, with higher values indicating greater usage of optimal codons and often predicting higher expression efficiency. The Effective Number of Codons (ENC) ranges from 20 to 61 and measures the degree of codon usage bias. Lower ENC values indicate stronger bias, whereas higher values indicate more random codon usage. The RSCU describes the usage frequency of a particular synonymous codon relative to a random distribution. An RSCU value greater than 1 indicates a codon is preferentially used; an RSCU value equal to 1 implies no obvious preference; and a value less than 1 indicates relatively infrequent usage. The Grand Average of Hydropathy (GRAVY) is an index used to measure the overall hydrophobicity or hydrophilicity of a protein. It is calculated by averaging the hydropathy scores of each amino acid in the protein sequence. A positive GRAVY value indicates overall hydrophobicity, while a negative value indicates overall hydrophilicity. GRAVY is commonly used to predict protein solubility and potential interactions with water molecules. Aromaticity (AROMO) reflects the proportion of aromatic amino acids (phenylalanine, tyrosine, and tryptophan) in a protein sequence. It is computed by dividing the number of aromatic amino acids by the total number of amino acids. Aromatic amino acids play important roles in protein stability and function, including ligand binding. All these values were calculated using CodonW 1.4.2 and the CAIcal server (Puigbo et al. 2008). Based on these metrics, we further performed neutrality plot analysis (GC12 vs. GC3), ENC‐GC3s plot analysis, PR2‐bias plot analysis, and optimal codon analysis, and visualized the results with R 4.4.2.

### 2.4 Phylogenetic Analysis

Eighteen complete mitochondrial genomes were used for phylogenetic analyses, covering two subfamilies and six genera of Engraulidae. Among these, the mitochondrial genomes of *Coilia mystus* , *Engraulis japonicus* , and *Setipinna taty* were previously sequenced by our research group and uploaded to the NCBI database. *Sardinops sagax* and *Ilisha elongata* were chosen as the outgroup.

Using PhyloSuite v1.2.3, we extracted the PCGs and RNA genes (RNAs) from the mitochondrial genome. Both PCGs and RNAs were aligned using MAFFT (Katoh and Standley 2013) under the “FFT‐NS‐1 (fast)” strategy. Gblocks and trimAI (Capella‐Gutierrez et al. 2009) were then employed to remove misaligned and low‐quality sites. The resulting gene sequences were concatenated into five datasets: (1) PCGs: Consists of 13 PCGs, totaling 11,388 sites. (2) PCGs12: Consists of the same 13 PCGs but excludes the third codon position, totaling 7592 sites. (3) PCGsRNA: Consists of 13 PCGs, 22 tRNAs, and 2 rRNAs, totaling 15,549 sites. (4) PCGs12RNA: Consists of 13 PCGs (with the third codon position removed), 22 tRNAs, and 2 rRNAs, totaling 11,753 sites. (5) Entire mitochondrial genome (Mt): Treats all sequences in the mitochondrial genome as a complete dataset, totaling 16,720 sites.

Phylogenetic trees were constructed using both maximum likelihood (ML) and Bayesian inference (BI) methods (Huelsenbeck and Ronquist 2001; Felsenstein 1981; Guindon and Gascuel 2003). ModelFinder v2.2.0 (Kalyaanamoorthy et al. 2017) was employed under the Bayesian information criterion (BIC) to identify the optimal partitioning strategy and evolutionary model for each dataset. The ML analyses were carried out in IQ‐TREE v2.2.0 (Lam‐Tung et al. 2015), whereas the BI analyses were conducted in MrBayes v3.2.7 (Ronquist et al. 2012). Two independent runs were performed for five million generations, sampling every 1000 generations; each run consisted of four Markov chains (three heated and one cold). The first 25% of sampled trees were discarded as burn‐in to mitigate potential bias. The BI analysis was deemed convergent once the average standard deviation of split frequencies (ASDSF) fell below 0.01. Finally, FIGtree was used to visualize and refine the resulting phylogenetic trees.

## 3 Rusults

### 3.1 General Characteristics of the Mitochondrial Genome

**FIGURE 1:**
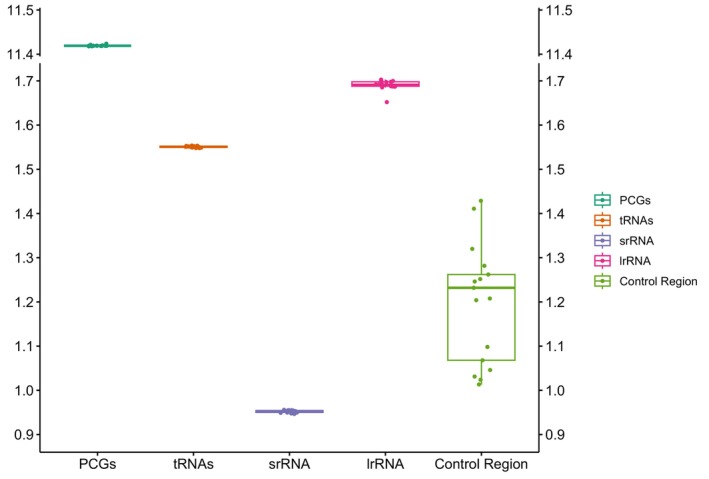
Sizes of protein‐coding genes (PCGs), tRNAs, large subunit rRNA (lrRNA), small subunit rRNA (srRNA), and control region in anchovies (family Engraulidae), unit (kilobase pairs).

We reassembled and reannotated the complete mitochondrial genomes of 18 common anchovy species (Engraulidae) from seas around China (including the previously uploaded genomes for *Setipinna taty* , *Engraulis japonicus* , and *Coilia mystus* ), and generated circular maps of their complete mitochondrial genomes (Figure S1). All anchovy mitochondrial genomes are the typical circular double‐stranded molecules, with lengths ranging from 16,665 to 17,125 bp. Based on gene region sizes, the PCGs, transfer RNAs (tRNAs), and ribosomal RNAs (rRNAs) show limited variation in length, whereas the control region exhibits considerable differences (Figure 1). Each anchovy genome contains 37 genes (including PCGs, tRNAs, rRNAs, the control region, and the L‐strand replication origin). Most of the coding genes (28) reside on the heavy (H) strand, while NAD6 and eight tRNA genes are located on the light (L) strand. The 12S and 16S rRNA genes lie between tRNAPhe (GAA) and tRNALeu (TAA), the control region is located between tRNAPro (TGG) and tRNAPhe (GAA), and the replication origin of the light strand (OL) is situated between tRNAAsn and tRNACys.

**FIGURE 2:**
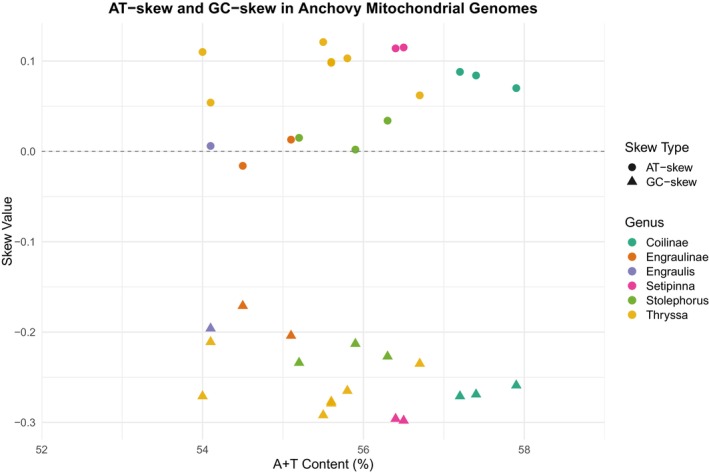
A + T content (%), AT skew, and GC skew in the mitochondrial genomes of 18 anchovy species (family Engraulidae). Species belonging to the same genus are represented by the same color.

In the 18 anchovy mitochondrial genomes, the A + T content ranges from 54% to 58%, consistently displaying a marked A + T bias, characterized by positive AT skew and negative GC skew (Figure 2). Within the PCGs, the base composition at each codon position reveals that the first codon position has a relatively lower A + T content, whereas the second and third positions are considerably higher, with the third position typically being the highest. The second codon position exhibits a strongly negative AT skew, while the first and third positions show a positive AT skew, resulting in an overall neutral AT skew across the entire PCG set. By contrast, the GC skew at the second and third positions (as well as for the entire PCGs) is strongly negative.

### 3.2 Codon Analysis

#### 3.2.1 Codon Usage Analysis

Across the 18 complete mitochondrial genomes, the total length of the 13 PCGs ranges from 11,418 to 11,424 bp. The average length of each gene is between 168 and 1836 bp, with *ATP8* being the shortest gene and *NAD5* being the longest. Among the 13 PCGs, 8 maintain the same length across different species. All PCGs initiate with the start codon ATG, except *COX1*, which uses GTG. The observed stop codons include T, TA, TAA, and TAG, with TAA being the most commonly used complete stop codon (Table S2).

**FIGURE 3:**
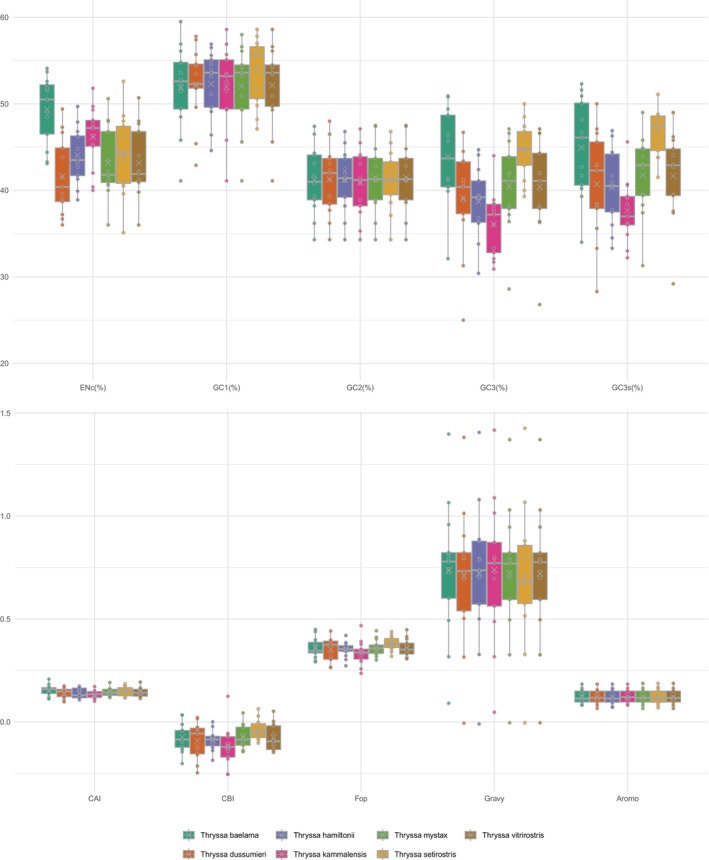
Codon usage indices for the protein‐coding genes (PCGs) of seven *Thryssa* species. CAI, Codon Adaptation Index; CBI, Codon Bias Index; ENC, Effective Number of Codons; FOP, Frequency of Optimal Codons.

Focusing on seven species of the genus *Thryssa*, the average GC1, GC2, and GC3 contents are 52.35%, 41.27%, and 40.50%, respectively. The average GC3s value of 41.97% indicates a preference for codons ending in A or T. Correspondingly, A3s, T3s, G3s, and C3s values are 49.77%, 26.10%, 13.10%, and 36.47%, respectively, implying that codons are most likely to end with A, followed by C, T, and G. The CAI across these genes ranges from 0.098 to 0.208, with *NAD4L* having the lowest CAI and *COX2* having the highest. Among the seven *Thryssa* species, Thryssa baelama shows the lowest CAI, whereas *Thryssa baelama* exhibits the highest. The CBI spans −0.254 to 0.125, with *NAD4L* at the lowest and *NAD3* at the highest; likewise, *Thryssa* has the lowest CBI and *Thryssa setirostris* the highest. The 13 PCGs have average Fop values ranging from 0.286 to 0.397, with *NAD6* being the lowest and *COX3* the highest. Among species, *Thryssa kammalensis* exhibits the lowest Fop value, whereas *Thryssa setirostris* shows the highest. All PCGs display positive GRAVY values, indicating these proteins are likely hydrophobic. The Aromo values range from 0.049 to 0.149, with *NAD2* being the lowest and *COX3* the highest (Figure 3).

#### 3.2.2 Codon Correlation Analysis

**FIGURE 4:**
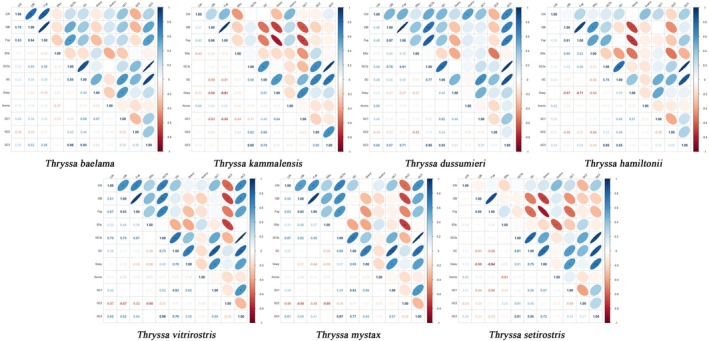
Pearson correlation heatmap of codon usage indices in different *Thryssa* species. This heatmap illustrates the Pearson correlation coefficients among various codon usage indices in the mitochondrial genomes of seven *Thryssa* species. Blue indicates a positive correlation, while red indicates a negative correlation. An asterisk indicates a statistically significant correlation at *p < 0.05, **p < 0.01, and ***p < 0.001, respectively. From left to right, the species are: *T. baelama* , *T. kammalensis* , *T. dussumieri* , *T. hamiltonii* , *T. vitrirostris* , *T. mystax* , *T. setirostris* .

In the seven examined *Thryssa* species, a significant correlation was observed between CBI and FOP (*p* < 0.001). Moreover, GC3s showed a significant correlation with overall GC content (*p* < 0.05), influencing codon usage bias in three *Thryssa* species, namely *Thryssa dussumieri* , *Thryssa vitrirostris* , and *Thryssa mystax* (Figure 4).

#### 3.2.3 Neutrality Plot Analysis

**FIGURE 5:**
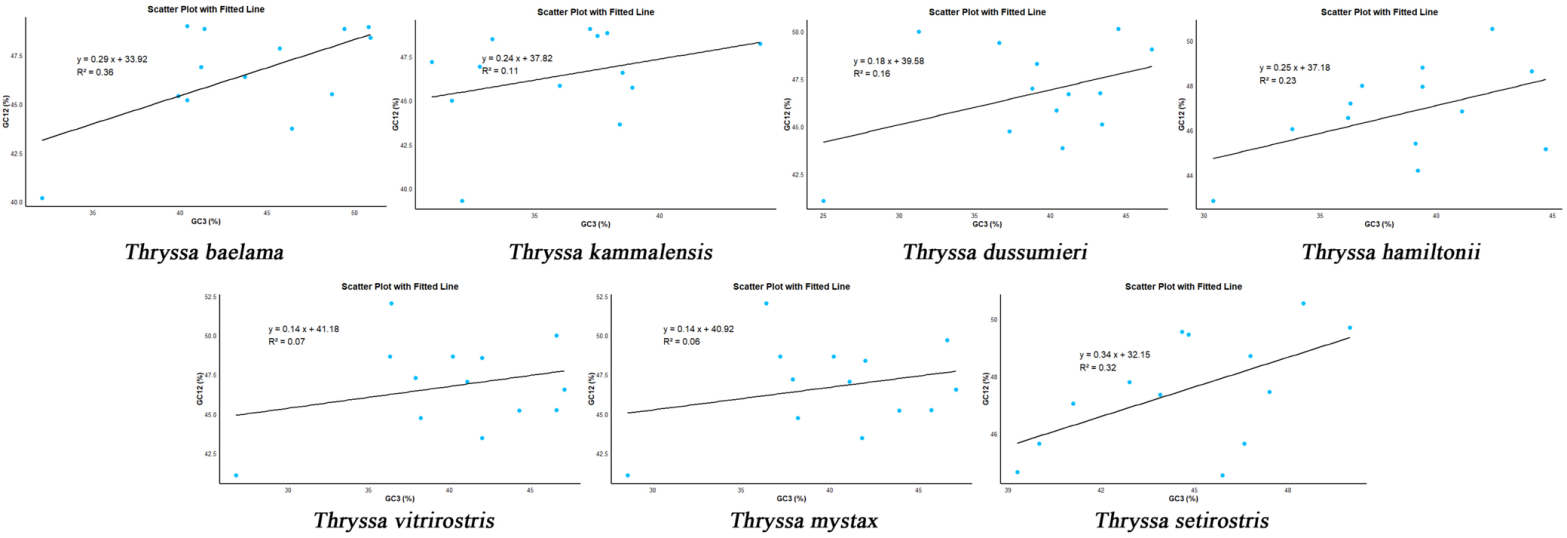
Neutrality plot analysis of GC content at the first two codon positions (GC12) versus the third codon position (GC3) in seven *Thryssa* species. From left to right, the species are: *T. baelama* , *T. kammalensis* , *T. dussumieri* , *T. hamiltonii* , *T. vitrirostris* , *T. mystax* , *T. setirostris* .

Based on the neutrality plot analysis, we investigated the relationship between GC content at the first two codon positions (GC12) and the third codon position (GC3). The GC12 content ranged from 40% to 52.55%, while GC3 ranged from 25.5% to 50.9%. The regression coefficient spanned from 0.14 to 0.34, with (*R* ^2^) values between 0.06 and 0.36, suggesting a very weak positive correlation between GC12 and GC3 (Figure 5). Statistical tests showed no significant correlation between GC12 and GC3, indicating that natural selection plays a predominant role in the codon usage bias observed in *Thryssa* mitochondrial genomes.

#### 3.2.4 ENC‐GC3s Plot Analysis

**FIGURE 6:**
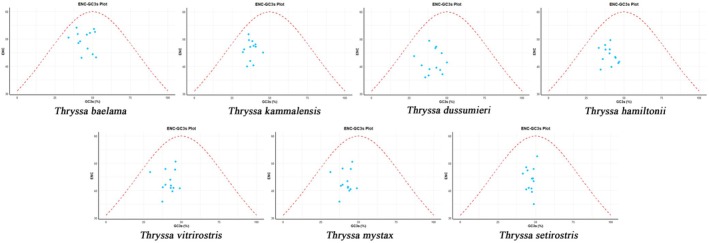
ENC‐GC3 plot analysis of PCGs from seven species of the genus *Thryssa*. The red dashed line represents the expected curve when codon usage bias is solely influenced by mutational pressure. From left to right, the species are: *T. baelama* , *T. kammalensis* , *T. dussumieri* , *T. hamiltonii* , *T. vitrirostris* , *T. mystax* , *T. setirostris* .

For all *Thryssa* species, the average ENC value exceeded 35, indicating relatively weak codon usage bias. In the ENC plot, the expected ENC curve represents the theoretical relationship between ENC values and GC3s under the sole influence of mutational pressure. All points fell below the expected ENC curve (Figure 6), implying that factors beyond mutational pressure, such as natural selection, also contribute to driving codon usage bias.

#### 3.2.5 PR2 Bias Plot Analysis

**FIGURE 7:**
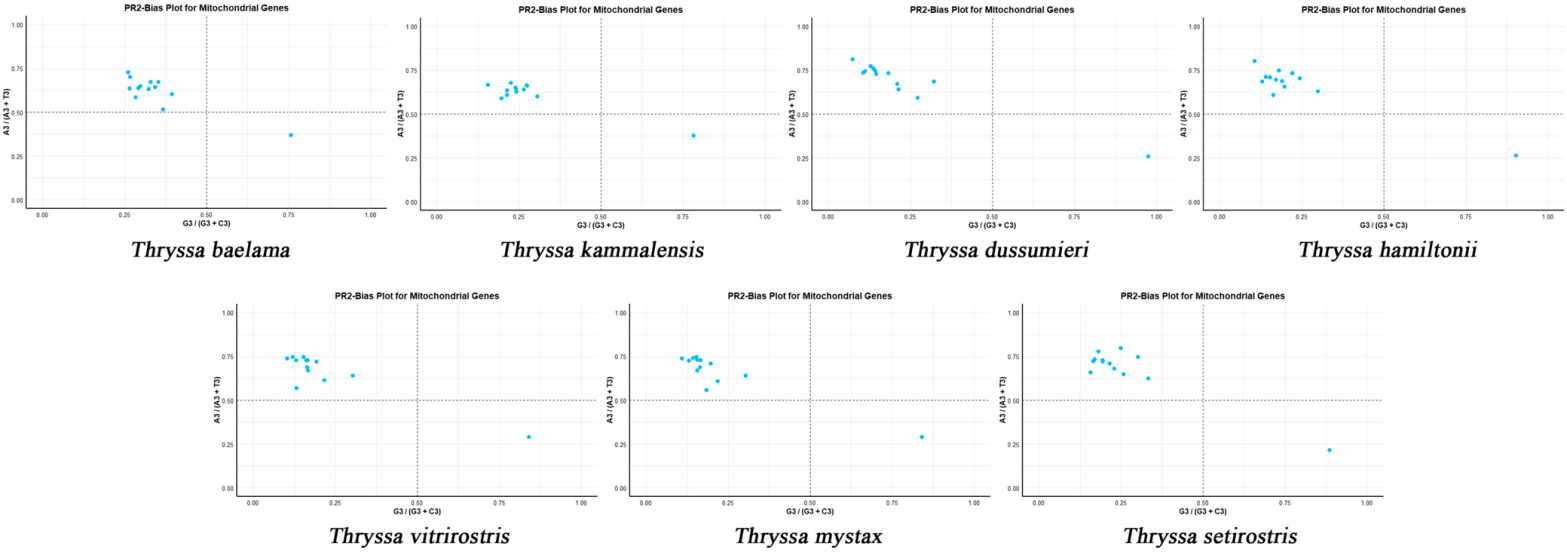
PR2 plot analysis of mitochondrial genome PCGs from seven species of the genus *Thryssa*. From left to right, the species are: *T. baelama* , *T. kammalensis* , *T. dussumieri* , *T. hamiltonii* , *T. vitrirostris* , *T. mystax* , *T. setirostris* .

We performed a parity rule 2 (PR2) bias plot analysis to investigate potential directional biases in the mitochondrial genes of *Thryssa* species. Both axes were set at 0.5, dividing the plot into four quadrants. The results showed that most data points clustered in the second quadrant, with five out of six genes not falling into the third quadrant. One exception (*NAD6*) was located in the fourth quadrant (Figure 7). These findings indicate a strong preference for A and C at the third codon position in *Thryssa*. Additionally, none of the seven species were distributed in the first quadrant (favoring A and G), suggesting a marked codon usage bias in the genus *Thryssa*.

#### 3.2.6 Analysis of Optimal Codons

**FIGURE 8:**
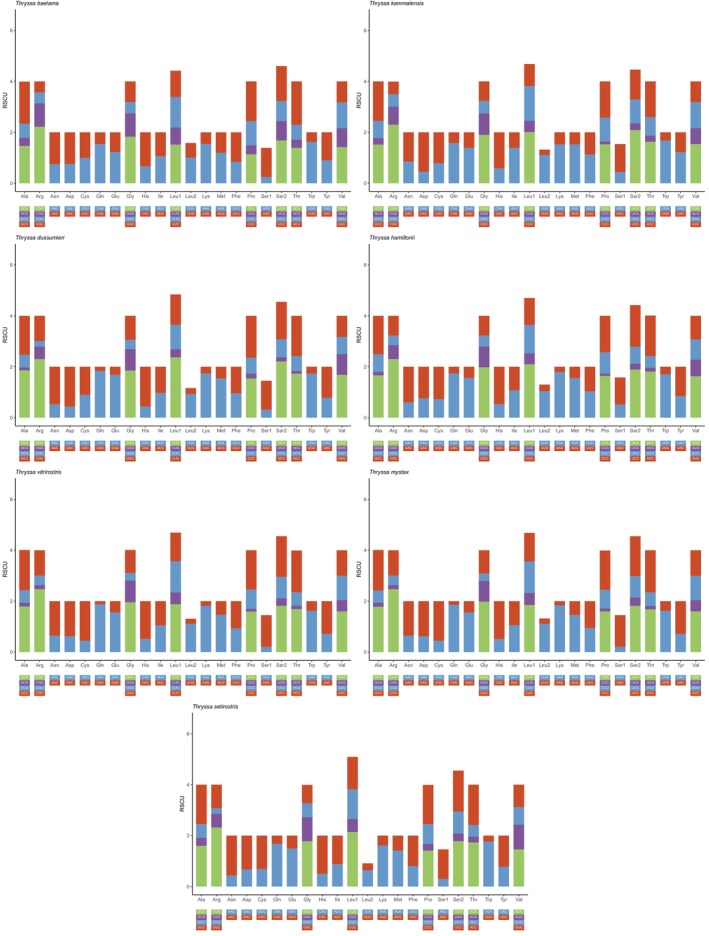
Relative synonymous codon usage (RSCU) analysis of 13 mitochondrial protein‐coding genes (PCGs) from seven species of the genus *Thryssa*. The colored blocks at the bottom represent different codons, with stop codons not included.

**FIGURE 9:**
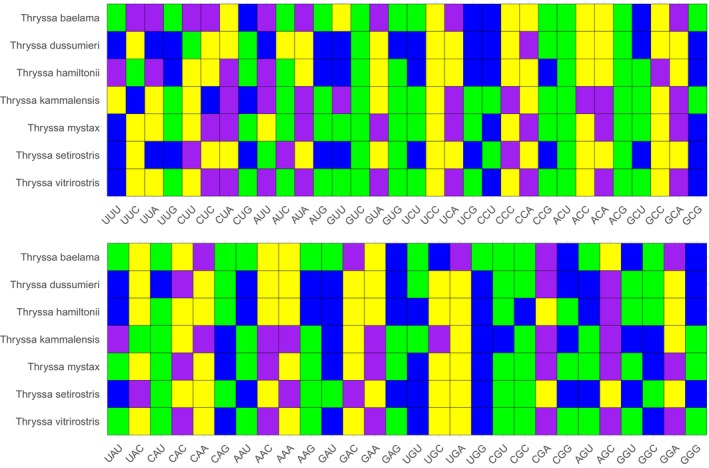
Optimal codons (ΔRSCU > 0.08, RSCU > 1) are marked in purple, high‐expression codons (ΔRSCU > 0.08) are marked in blue, and high‐frequency codons (RSCU > 1) are marked in yellow for seven species of the genus *Thryssa*.

RSCU analysis of seven *Thryssa* species revealed a total of 32 high‐frequency codons (HFCs) (Figure 8). Among these species, 28 HFCs were identified in *T. baelama* , *T. kammalensis* , *T. hamiltonii* , *T. vitrirostris* , and *T. mystax* ; 26 in *T. dussumieri* ; and 27 in *T. setirostris* . Of the 32 HFCs, 14 ended with A, 13 with C, and 5 with T, indicating a preference for codons ending in A or C. Across the seven species, the numbers of highly expressed codons (∆RSCU > 0.08) were 23, 26, 24, 27, 22, 26, and 23, respectively. The numbers of optimal codons (ΔRSCU > 0.08 and RSCU > 1) were 14, 4, 6, 17, 14, 6, and 15, respectively. Among all 31 optimal codons, 14 ended in A, 12 ended in C, and 5 ended in T. The most frequently used codons are CGA and AGC, each identified as optimal codons in five species. Next are AUA, AUU, CUA, GCA, and UCA, each identified as optimal codons in four species. Apart from AGC, these codons predominantly end with A or T, indicating a preference for A/T‐ending codons in the genus *Thryssa*. Additionally, the optimal codons of *Thryssa mystax* and *Thryssa vitrirostris* are very similar, differing only by the codon AUU, suggesting a close evolutionary relationship (Figure 9).

### 3.3 Nucleotide Diversity and Evolutionary Rate

Nucleotide diversity (*P*_i_) measures the average difference between two randomly selected nucleotide sequences within a population, serving as a crucial parameter in population genetics to assess genetic diversity. Among the 13 PCGs of the seven *Thryssa* species, *NAD2*, *NAD3*, and *NAD6* exhibit the highest nucleotide diversity (*P*_i_), whereas *COX1*, *COX2*, and *COX3* display the lowest *P*_i_ values (Table 1).

**TABLE 1:** Nucleotide diversity (*P*_i_), average K2P pairwise genetic distance, and average *K*_a_/*K*_s_ ratio for seven species of the genus *Thryssa*.

Gene	*P*_i_	K2P (average)	*K*_a_/*K*_s_ (average)
*ATP6*	0.1715	0.2008	0.0218
*ATP8*	0.1596	0.1883	0.0433
*COX1*	0.1483	0.1704	0.0037
*COX2*	0.1343	0.1531	0.0060
*COX3*	0.1326	0.1496	0.0101
*CYTB*	0.1665	0.1931	0.0100
*NAD1*	0.1634	0.1894	0.0163
*NAD2*	0.1974	0.2360	0.0704
*NAD3*	0.1964	0.2370	0.0364
*NAD4*	0.1788	0.2105	0.0321
*NAD4L*	0.1673	0.1966	0.0301
*NAD5*	0.1765	0.2077	0.0403
*NAD6*	0.1790	0.2119	0.0633

To gain deeper insights into evolutionary rates, we calculated the ratio of nonsynonymous (*K*_a_) to synonymous (*K*_s_) substitutions (*K*_a_/*K*_s_) for each of the 13 PCGs as an indicator of selective pressure. All *K*_a_/*K*_s_ values were below 1, suggesting that these genes are under purifying selection. Within this range, *COX1* and *COX2* have the lowest *K*_a_/*K*_s_ values, while *NAD2* and *NAD6* show relatively higher *K*_a_/*K*_s_ values.

Pairwise genetic distances (K2P) based on the 13 PCGs were computed for the seven *Thryssa* species. *COX1*, *COX2*, and *COX3* exhibit the lowest mean K2P distances, whereas *NAD2* and *NAD3* have the highest pairwise genetic distances.

### 3.4 Phylogenetic Analysis

**FIGURE 10:**
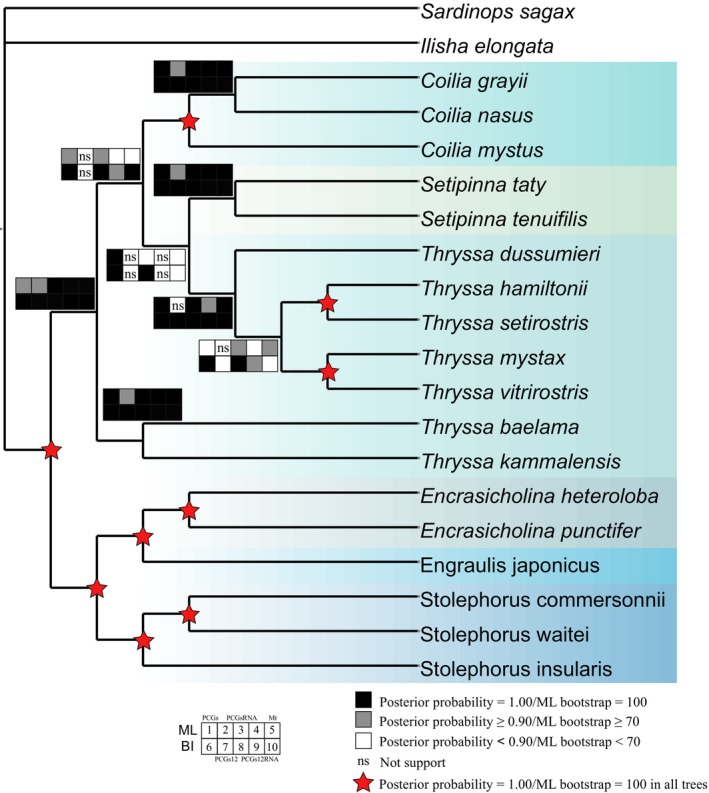
Reconstructed phylogenetic tree of the family Engraulidae based on phylogenetic analysis. Adopting the topology with higher support; see the legend for node support values and dataset sources.

We performed phylogenetic analyses using five datasets, with the *Sardinops sagax* (Clupeidae) and the *Ilisha elongata* (Pristigasteridae)as the outgroup (Figure 10; Figure S2). Both ML and BI methods were employed to construct phylogenetic trees. Our results support the monophyly of Engraulidae and confirm the non‐monophyly of the genus *Thryssa* across all analyses, highlighting variability in the phylogenetic placement of genus *Setipinna*. Consistent topologies were recovered in the six trees based on mitochondrial genome (Mt), PCGs, and PCGsRNA datasets. The subfamilies Engraulinae and Coiliinae formed two distinct branches, with four genera (excluding *Thryssa*) consistently supported as monophyletic. Within Engraulinae, *Stolephorus* diverged first (posterior probability = 1/1/1/1/1; ML bootstrap = 100/100/100/100/100), while *Encrasicholina* and *Engraulis* grouped together (posterior probability = 1/1/1/1/1; ML bootstrap = 100/100/100/100/100) (Figure 10). Within Coiliinae, *T. baelama* and *T. kammalensis* formed a distinct clade diverging earliest in four analyses (posterior probability = 1/ns/1/1/1; ML bootstrap = 98/ns/100/100/100). The monophyly of genus *Coilia* was strongly supported (posterior probability = 1/1/1/1/1; ML bootstrap = 100/100/100/100/100). However, the phylogenetic position of the genus *Setipinna* varied across analyses. It clustered with the remaining lineage of *Thryssa* in the PCGs‐ML, PCGs‐BI, PCGsRNA‐ML, PCGsRNA‐BI, Mt‐ML, and Mt‐BI trees (posterior probability = 1/1/0.58; ML bootstrap = 72/53/63), whereas in analyses excluding third codon positions (PCGs12‐ML, PCGs12‐BI, PCGs12RNA‐ML, PCGs12RNA‐BI), it was identified as a sister group to *Coilia* (posterior probability = 0.97/1; ML bootstrap = 57/65).

## 4 Discussion

Based on the phylogenetic analysis using complete mitochondrial genomes, this study reconstructed the phylogenetic relationships within the family Engraulidae and confirmed the non‐monophyletic status of the genus *Thryssa*. Additionally, the analysis of codon usage bias in seven species of *Thryssa* further supported the significant role of natural selection in their evolutionary processes.

### 4.1 Characteristics of the Mitochondrial Genome

Comparative analyses show that Engraulidae mitogenomes are highly conserved in structure, consistent with findings in other teleost fishes (Catanese et al. 2010; Liu et al. 2022; Lavoue et al. 2007). Differences in gene length primarily reside in the control region (Brzuzan 2000), whose remarkable length variation may be related to its roles in replication and transcriptional regulation, and it represents an important source of mitochondrial genomic diversity. The control region has been shown to evolve faster than other genes in fish mitogenomes (Maté et al. 2004), providing abundant molecular markers for population genetics and phylogenetic research. All 18 anchovy mitogenomes exhibit a marked A + T bias in nucleotide composition, consistent with other Clupeiformes (Cui et al. 2018). The third codon position has the highest A + T content, likely because this position is degenerate and can accumulate more mutations during evolution.

### 4.2 Codon Usage Bias

Through analysis of genetic information, we found that synonymous codon usage varies among species, with certain codons used more frequently than others. Codon usage bias is influenced by multiple factors, including base composition, gene length, expression level, tRNA abundance, amino acid hydrophobicity, aromaticity, mutation, and selection (Chen et al. 2014; Trotta 2013), with mutation and selection often playing the most critical roles.

To investigate the genetic structure and evolutionary trends of seven *Thryssa* species, we examined their codon usage bias. The 13 PCGs show little variation in length among species, reflecting a high degree of conservation. Some genes feature incomplete stop codons (T, TA), a typical mitochondrial characteristic wherein post‐transcriptional modification can yield the complete stop codon TAA; this mechanism contributes to genome compactness by reducing noncoding intergenic regions (Bratic et al. 2016). Analysis of GC content indicates a preference for A or T at the third codon position, aligning with common patterns in eukaryotic mitochondrial codon usage (Tang et al. 2022). The values of A3s, T3s, G3s, and C3s reinforce this finding. Such preferences may be related to tRNA abundance and the pairing efficiency of anticodons. Variations in CAI and CBI further reflect differences in codon usage among genes and species. Genes with higher CAI values (e.g., *COX2*) may be subject to stronger selection pressure or maintain higher expression levels. FOP values support these observations. Positive GRAVY values indicate that these encoded proteins are likely hydrophobic, consistent with the fact that many mitochondrial proteins participate in the electron transport chain and embed in membrane structures (Gao et al. 2024). Variation in aromatic amino acid content (Aromo) may affect protein structure and function; for instance, *COX3* shows the highest Aromo value, possibly linked to its specific role in electron transport. Correlations among codon base composition, GC3s, CAI, CBI, and FOP suggest that base composition significantly influences codon usage bias. Neutrality plot analysis and PR2‐bias analysis also indicated that natural selection plays a more prominent role in shaping codon usage bias within the genus *Thryssa*, consistent with mitochondrial genome findings in other organisms (Barbhuiya et al. 2021; Li et al. 2023).

In fact, codon usage bias is generally influenced by both mutational pressure and natural selection, but the relative importance of these two factors may vary among different study organisms (Chen et al. 2014; Trotta 2013). From the perspective of evolutionary history and taxonomic position, determining whether codon usage patterns observed within the genus *Thryssa* are unique requires comparison with additional data from other Clupeiformes. Previous studies have shown that many clupeiform fishes exhibit similar mitochondrial genomic characteristics, including an A + T bias at the third codon position and the frequent occurrence of incomplete stop codons (Cui et al. 2018; Lavoue et al. 2007), consistent with our findings. Sebastian et al. (2022), in their analysis of codon usage bias across 70 mitochondrial genomes of clupeids, concluded that mutation pressure was the primary factor influencing codon bias; however, their study included only one *Thryssa* species. In contrast, our research indicates that natural selection plays a more prominent role in shaping codon usage bias in *Thryssa*, possibly because our samples were all collected from the East China Sea and the South China Sea, reflecting adaptation to similar ecological environments. Currently, research on codon usage bias in Clupeiformes remains limited. Expanding sampling efforts to include more species within Clupeiformes would further clarify both common and unique evolutionary patterns of codon usage within *Thryssa*, providing deeper insight into the multiple evolutionary forces driving microevolution and phylogenetic relationships in this group.

### 4.3 Phylogeny of Engraulidae and Genus *Thryssa*

The phylogenetic analyses provided strong evidence supporting the monophyly of the family Engraulidae, with the 18 species classified into two subfamilies and six genera. These findings are consistent with previous morphological and molecular studies (Bornbusch and Lee 1992; Lavoue et al. 2013, 2010, 2017; Bloom and Lovejoy 2014). The division into Engraulinae and Coiliinae received robust support across all datasets analyzed (posterior probability = 1, ML bootstrap = 100). Among the six species of the subfamily Engraulinae, genus *Stolephorus* diverged first, while *Setipinna* and *Engraulis* formed sister groups, a relationship strongly supported across all datasets and consistent with previous research. Within the subfamily Coiliinae, *Coilia* and *Setipinna* were both recovered as monophyletic groups, genus *Thryssa* was found to be non‐monophyletic, with notable variations in intergeneric relationships. Specifically, in the four datasets that excluded the third codon positions, *Setipinna* grouped with *Coilia*, and this clustering received strong support in the PCGs12‐BI and PCGs12RNA‐BI datasets, aligning well with recent findings (Pang et al. 2019; Lewis and Lema 2019; Hu et al. 2019). Due to the degeneracy of the genetic code, third codon positions typically evolve faster, leading to saturation from multiple substitutions, potentially obscuring genuine phylogenetic signals. Removing these third codon positions reduces noise caused by saturation and stabilizes sequence comparisons. Saturation analyses performed on the four datasets (excluding the complete mitochondrial genome dataset) indicated significantly lower substitution saturation in datasets without third codon positions (Figure S3). Consequently, phylogenetic results derived from these reduced datasets are likely more reliable and better reflect the true evolutionary relationships.

Our results revealed two distinct lineages within the genus *Thryssa*. *T. baelama* and *T. kammalensis* , characterized by shorter maxillae extending only to the operculum, diverged first. The other lineage comprised *T. dussumieri* , *T. hamiltonii* , *T. mystax* , *T. setirostris* , and *T. vitrirostris* , differentiated mainly by the extent of the maxilla: reaching the posterior edge of the pectoral fin in *T. dussumieri* , extending to the anal opening in *T. setirostris* , and reaching the base of the pectoral fin in *T. mystax* and *T. vitrirostris* , with fewer than 17 lower gill rakers distinguishing *T. mystax* . In contrast, *T. hamiltonii* , with a maxilla extending to the gill opening, has traditionally been considered more closely related to *T. dussumieri* . Previous studies by Ma et al. (2010) and Zhang et al. (2016), based respectively on 16S rRNA and *COX1* gene sequences, grouped *T. dussumieri* and *T. hamiltonii* together. However, our analyses consistently demonstrated a closer phylogenetic relationship between *T. hamiltonii* and *T. setirostris* , strongly supported across all reconstructed phylogenetic trees and aligning with the findings of Ma et al. (2015). Additionally, our study revealed extremely low genetic distances between *T. vitrirostris* and *T. mystax* , consistent with previous molecular studies, none of which could clearly distinguish between these two taxa. Ma et al. (2010) suggested that they might represent a single species. Given these unresolved issues, the classification of the genus *Thryssa* remains problematic, and further comprehensive studies are needed to establish a more accurate taxonomic framework.

In the future, identifying suitable methods for species identification and reliable phylogenetic relationships within *Thryssa* will be one of the focuses of research. Among the most commonly used genes in fish identification is the *COX1* gene (Yang et al. 2019; Ceruso et al. 2019; Persis et al. 2009). However, the widely used *COX1* gene may have low nucleotide sequence divergence in certain taxonomic groups due to its slow evolution, which can hinder the identification of some closely related species (Cawthorn et al. 2015; Rubinoff et al. 2006; Terio et al. 2010; Ward et al. 2009; Zhang and Cheng 2012). Previous studies based on *COX1* have also shown that this gene has low phylogenetic resolution within *Thryssa*. Some studies suggest that multi‐gene barcoding, combining multiple gene fragments, might be a reliable approach (Zhang et al. 2016; Ma et al. 2015; Gangan and Pavan‐Kumar 2019). We calculated the *K*_a_/*K*_s_, *P*_i_, and K2P genetic distances of mitochondrial PCGs and found several potential mitochondrial genes with higher resolution. A multi‐gene barcode suitable for *Thryssa* species can be developed in the future, and providing more reliable genetic evidence for its phylogenetic position, its effectiveness remains to be validated by further research.

### 4.4 Limitations and Future Perspectives

This study provides new molecular evidence for the phylogeny and species identification of Engraulidae. Given the significant economic and ecological importance of this family in Chinese and adjacent marine areas, our results have practical implications for conservation and fisheries management. Firstly, accurately identifying species and determining their systematic positions are fundamental steps in distinguishing distinct fishery stocks. Molecular markers, especially for morphologically similar and easily misidentified groups such as *Thryssa*, can help uncover cryptic species and precisely assess population structure (Hebert et al. 2003). Secondly, employing complete mitochondrial genomes or other high‐resolution molecular markers for monitoring fisheries resources can help detect significant changes in population sizes or genetic diversity, thereby informing scientific management strategies and habitat restoration efforts (Helyar et al. 2011).

Nevertheless, our study has certain limitations. First, our sample coverage was relatively restricted, with limited species sample sizes and insufficient representation of population diversity and geographic variation. Future studies should include larger and more geographically diverse sample groups to further enhance the accuracy and robustness of phylogenetic inferences. Regarding methodology, while mitochondrial markers have been widely applied in species identification and molecular systematics, the absence of nuclear genomic information may overlook some evolutionary events. Therefore, future research should integrate nuclear genomic data or even transcriptomic and genome resequencing approaches, combined with multi‐gene barcoding and high‐throughput sequencing techniques such as RAD‐seq, ddRAD, and whole‐genome sequencing (WGS), to achieve a more refined understanding of the phylogenetic relationships within Engraulidae and the genus *Thryssa*.

## 5 Conclusion

In conclusion, this study provides comprehensive molecular evidence for understanding mitochondrial genome structure, codon usage bias, and phylogenetic relationships within the Engraulidae, particularly among species of the genus *Thryssa*. By analyzing complete mitochondrial genomes from 18 Engraulidae species sampled from waters around China, we found structural conservation of mitochondrial genomes and a pronounced A + T bias at third codon positions. Codon usage analyses of seven *Thryssa* species further demonstrated the significant role of natural selection in shaping their PCGs. Phylogenetic analyses reconstructed evolutionary relationships within Engraulidae, notably revealing that analyses excluding third codon positions yielded a closer relationship between *Setipinna* and *Coilia* than with *Thryssa*. Moreover, the genus *Thryssa* was confirmed as non‐monophyletic, clearly dividing into two distinct lineages. Although mitochondrial *COX1* is widely employed for fish species identification, its resolution within *Thryssa* is limited, underscoring the need for more suitable molecular markers. Future research combining multi‐gene barcoding, advanced high‐throughput sequencing technologies, and nuclear genomic data, along with expanded geographic sampling and larger sample sizes, will further refine phylogenetic and taxonomic frameworks within Engraulidae.

## Author Contributions

**Wenyu Sun:** formal analysis (lead), investigation (lead), visualization (lead), writing – original draft (lead). **Yibang Wang:** conceptualization (equal), writing – review and editing (equal). **Hui Zhang:** conceptualization (equal), funding acquisition (lead), project administration (lead), writing – review and editing (equal).

## Conflicts of Interest

The authors declare no conflicts of interest.

## Supporting information


Appendix S1.

Appendix S2.

## Data Availability

The raw data used in this article has been uploaded to the public database *figshare* (https://doi.org/10.6084/m9.figshare.28227167.v2).
