# Supplementary material for: Genetic Characteristics and Phylogenetic Relationships of 18 Anchovy Species Based on Mitochondrial Genomes in the Seas Around China
Source: Ecol Evol. 2025 May 24;15(5):e71496. doi: 10.1002/ece3.71496 (PMC12102761; doi:10.1002/ece3.71496)
Supplement: Supplementary file 1 — Appendix S1. [file ECE3-15-e71496-s001.docx]

**Supplementary Material:**

**Table S1. The usage of start and stop codons in 18 *Engraulidae* species.**

**
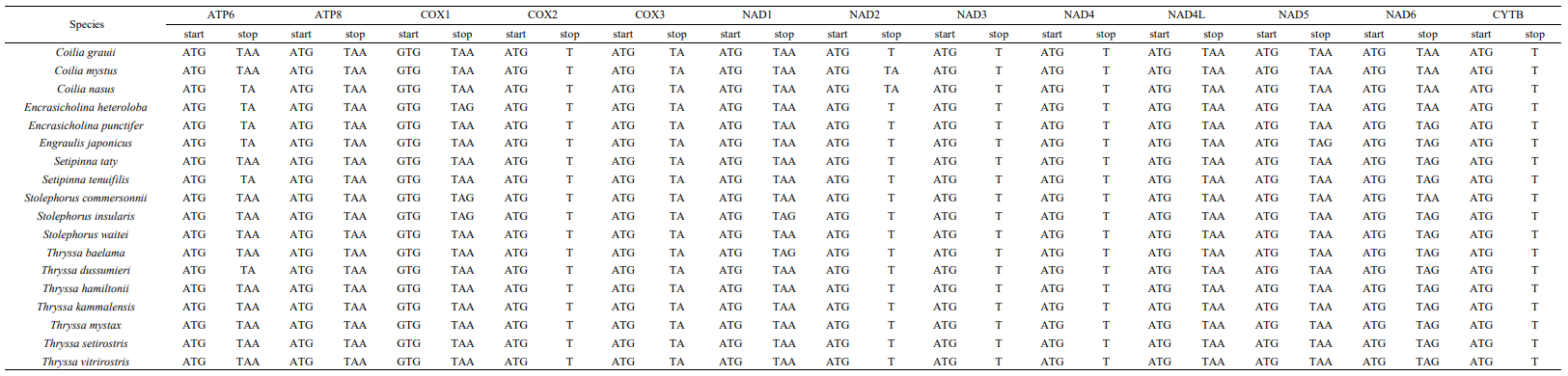
**

**Figure S1. Circular Map of the Mitochondrial Genome of Anchovies (Family Engraulidae)(a.** ***Thryssa baelama,* b*.*** ***Thryssa kammalensis,* c*.*** ***Thryssa dussumieri,* d*.*** ***Thryssa hamiltonii,* e*.*** ***Thryssa setirostris,* f. *Thryssa mystax,* g*.*** ***Thryssa vitrirostris,* h. *Coilia nasus,* i*. Coilia mystus,* j*. Coilia grayii,* k. *Setipinna tenuifilis,* l*. Setipinna taty,* m*. Encrasicholina heteroloba,* n*. Encrasicholina punctifer,* o*. Engraulis japonicus,* p*. Stolephorus commersonnii,* q*. Stolephorus insularis,* r*. Stolephorus waitei.*)**

**
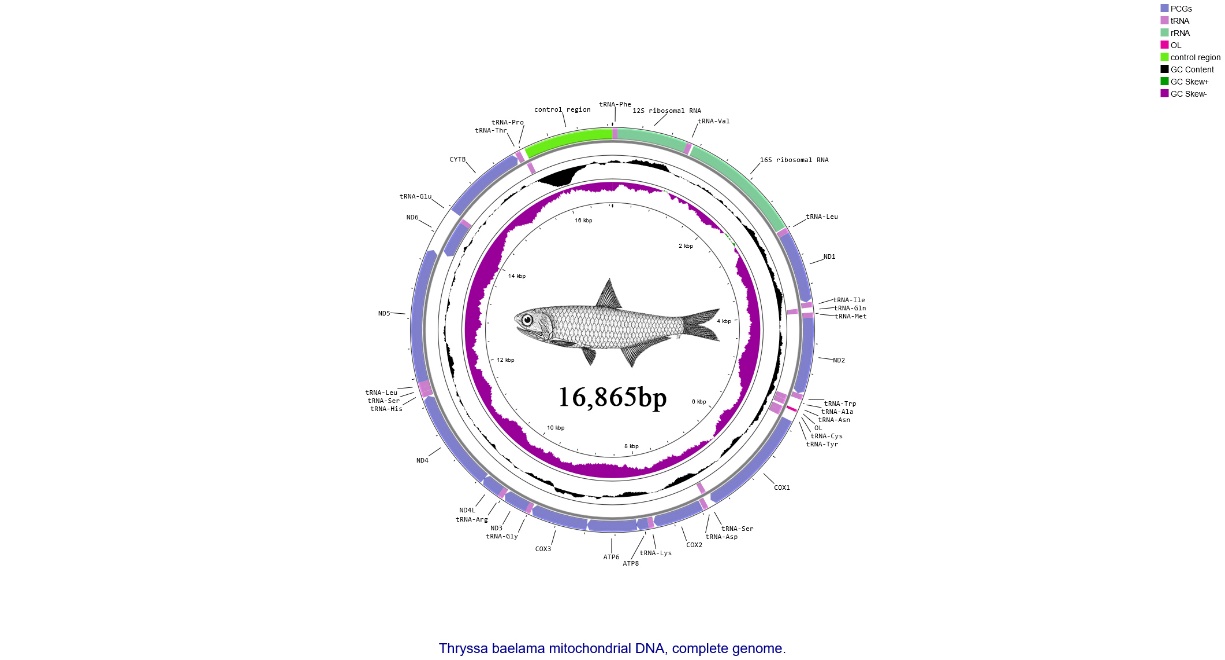
**

**a**

**
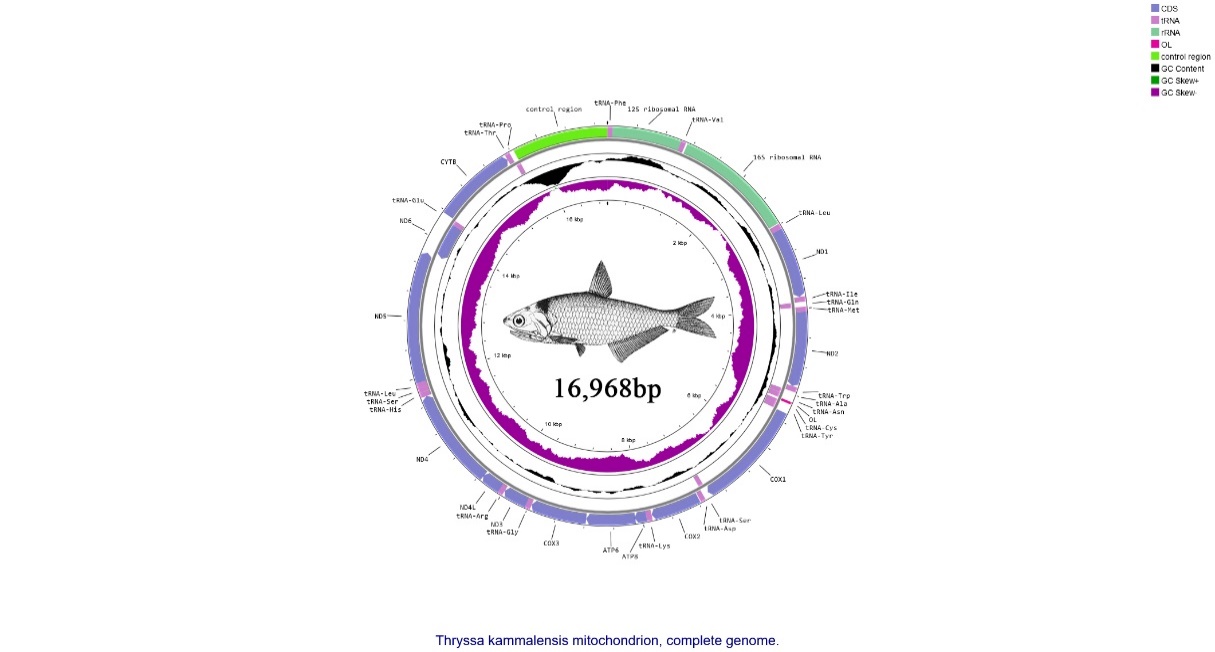

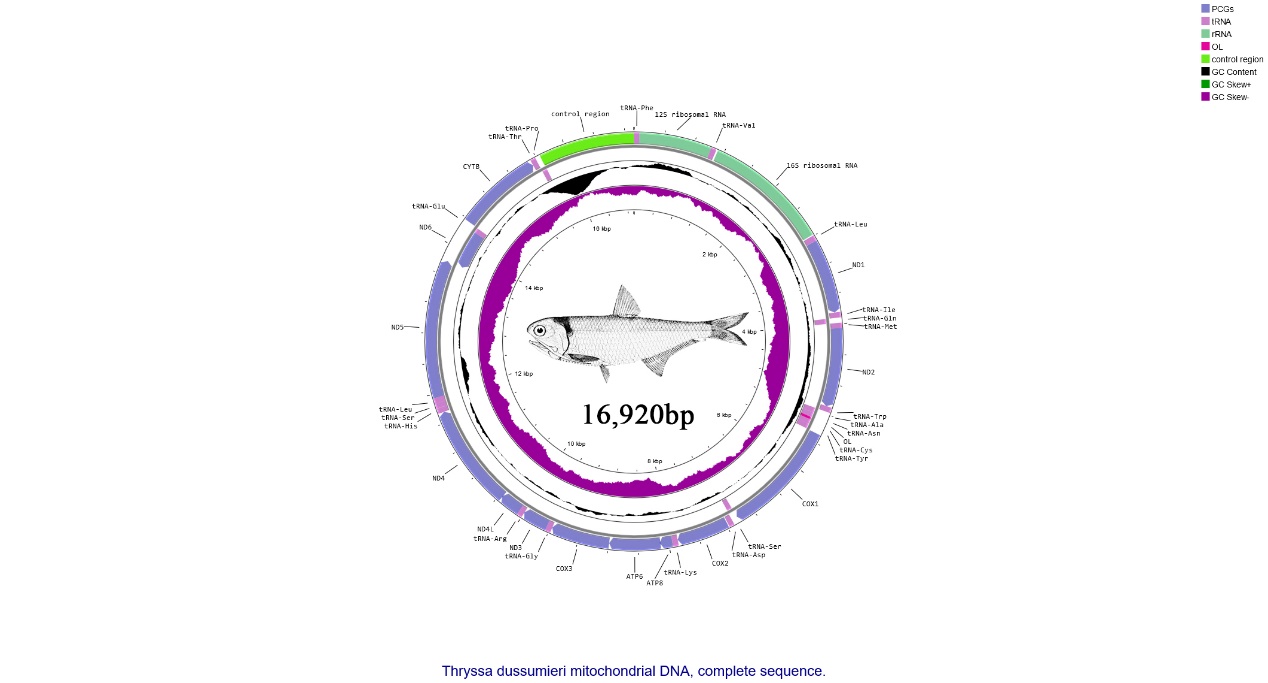

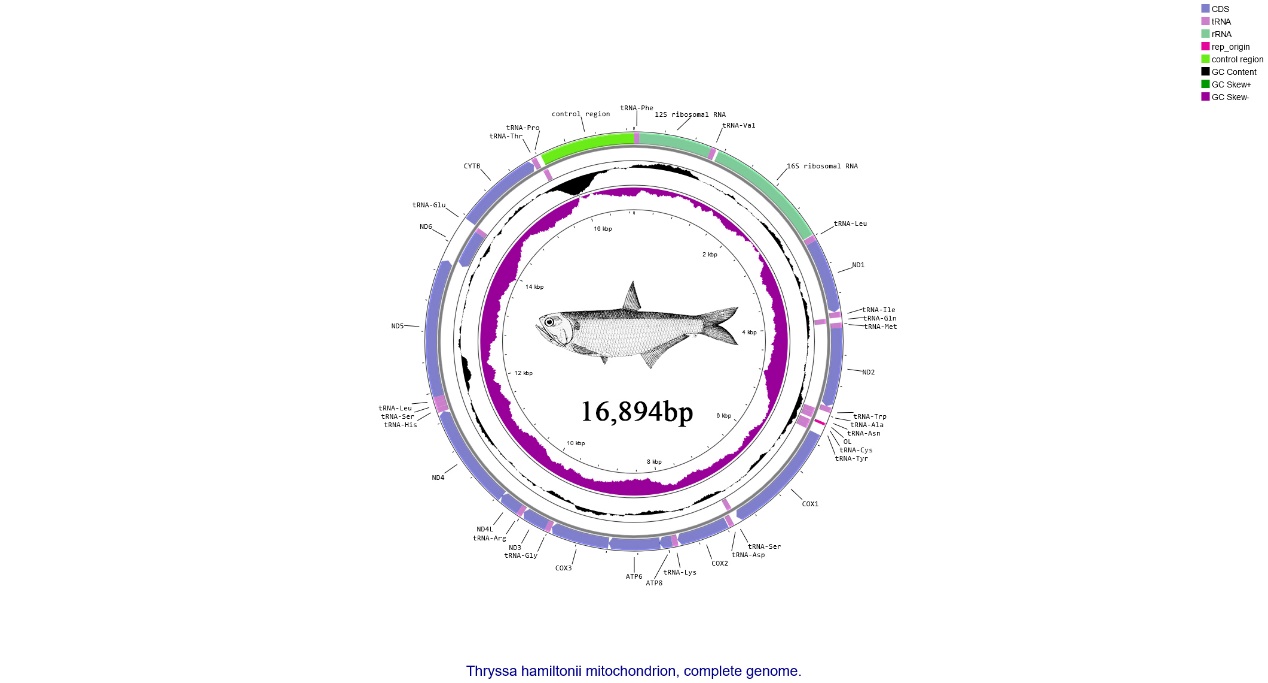

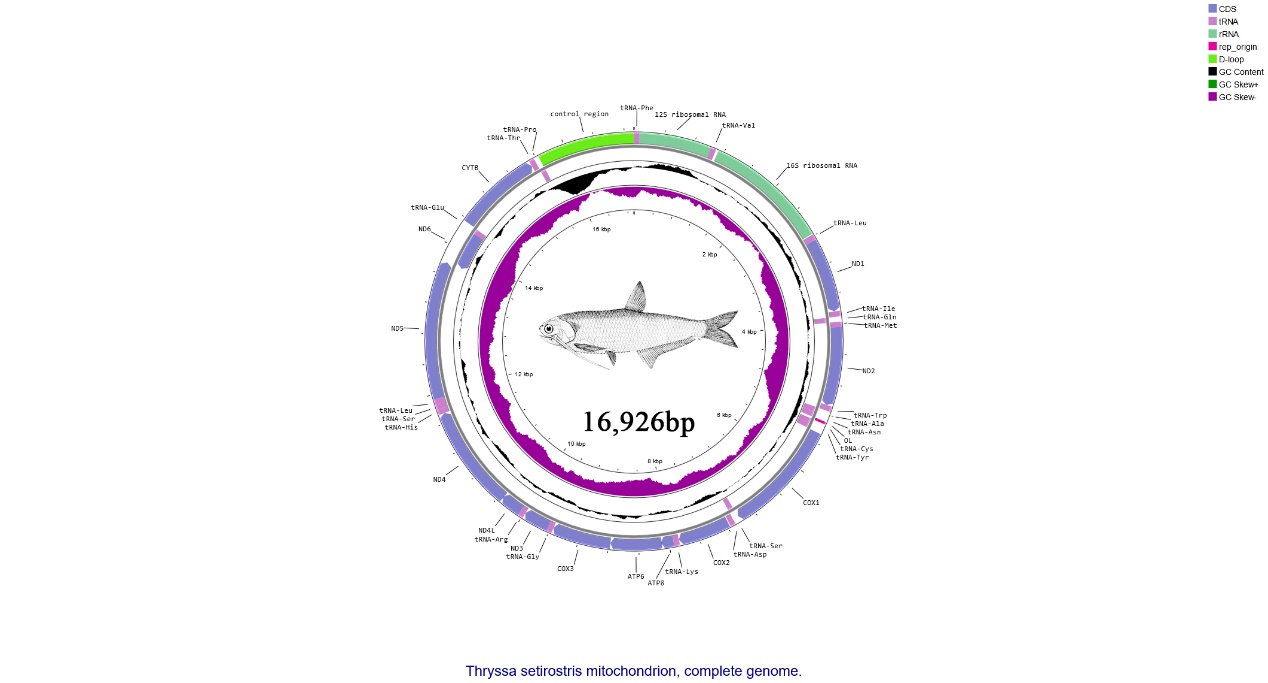

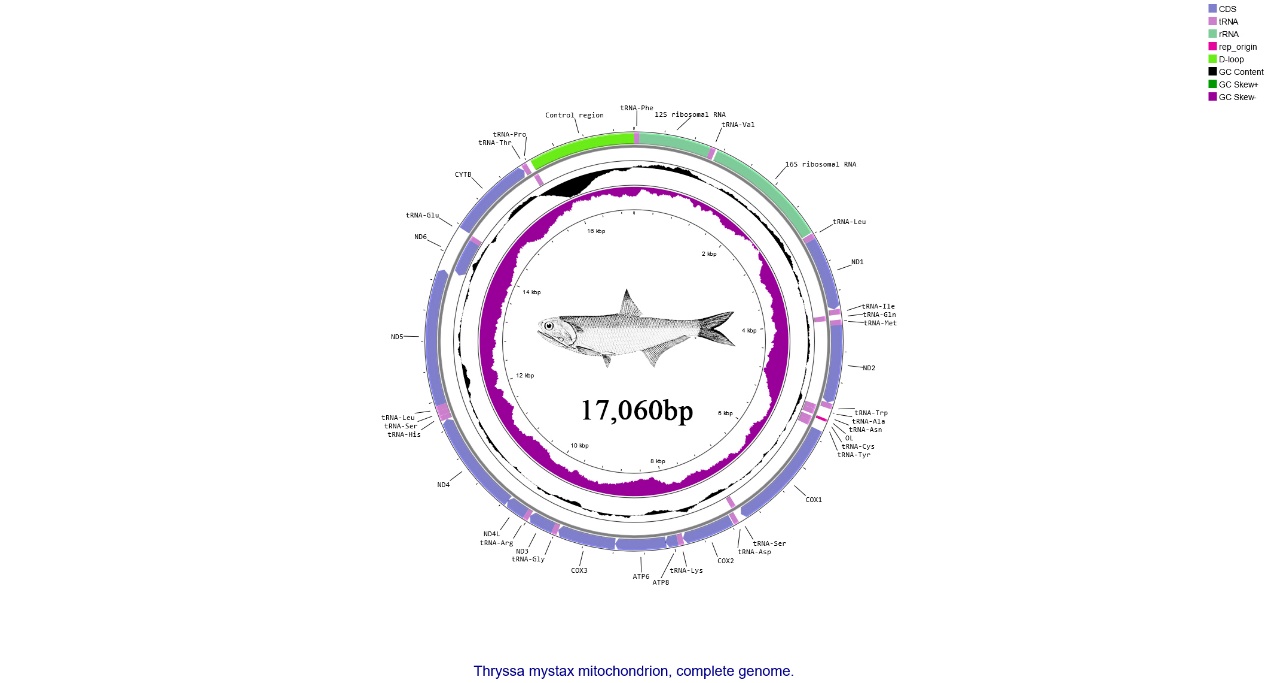
**

**b**

**c**

**d**

**e**

**f**

**
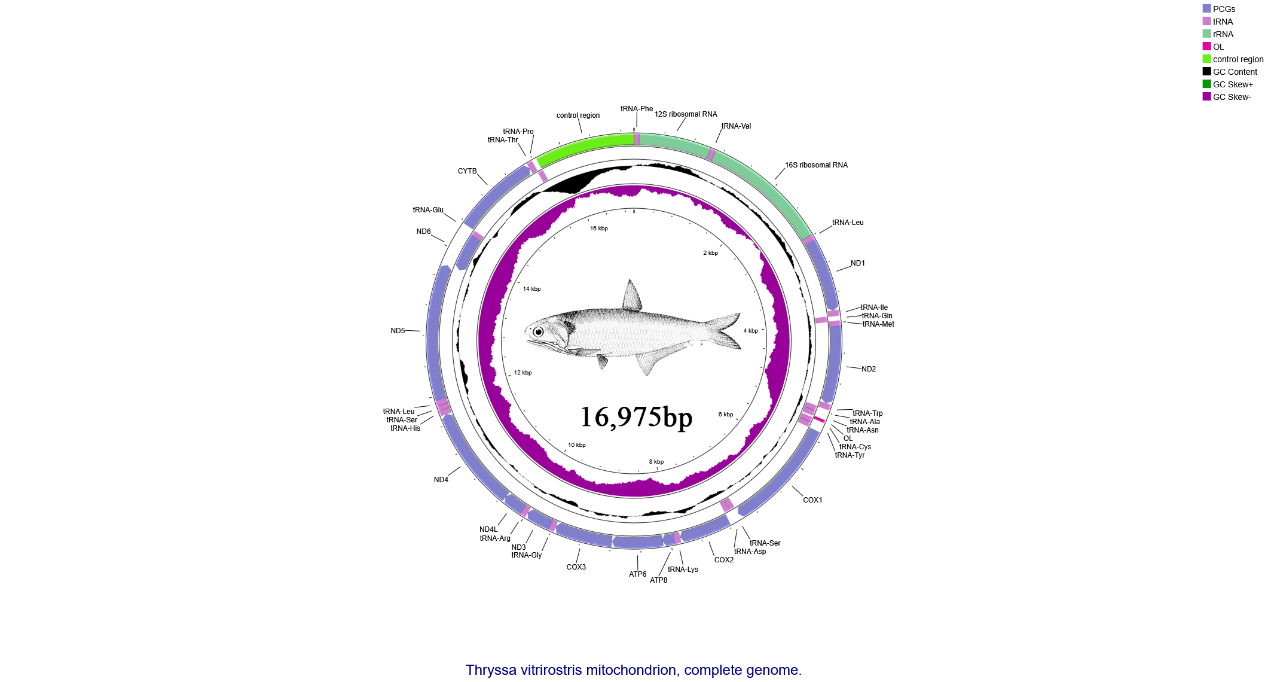
**

**g**

**
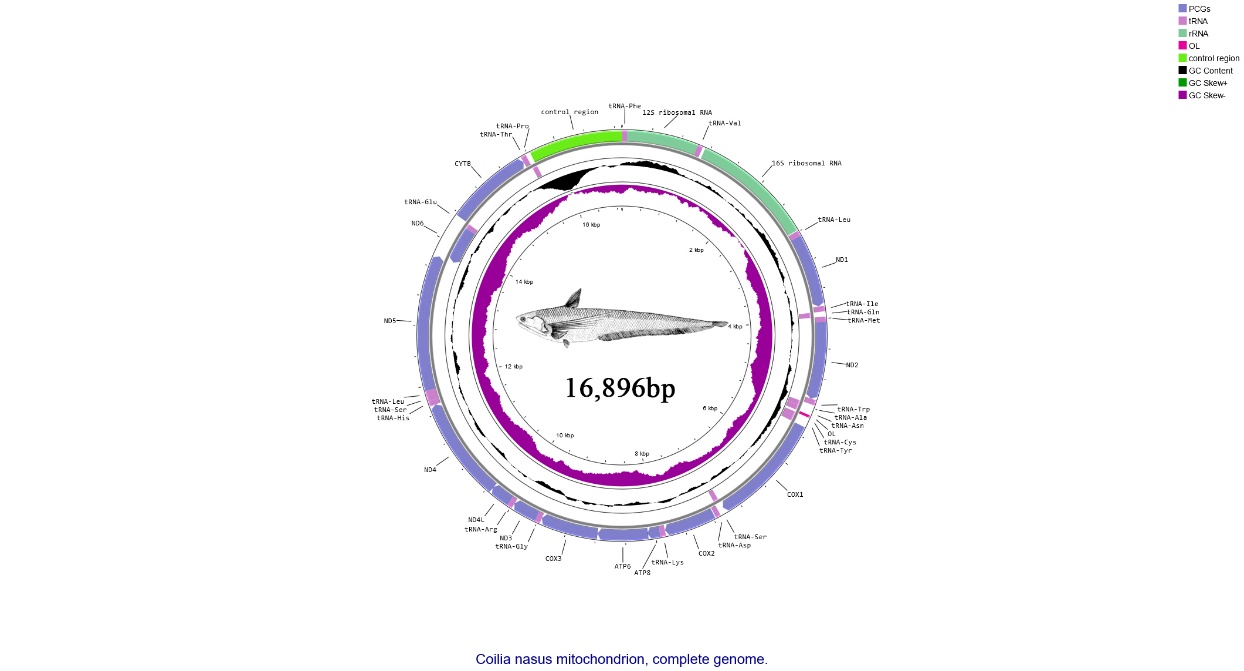

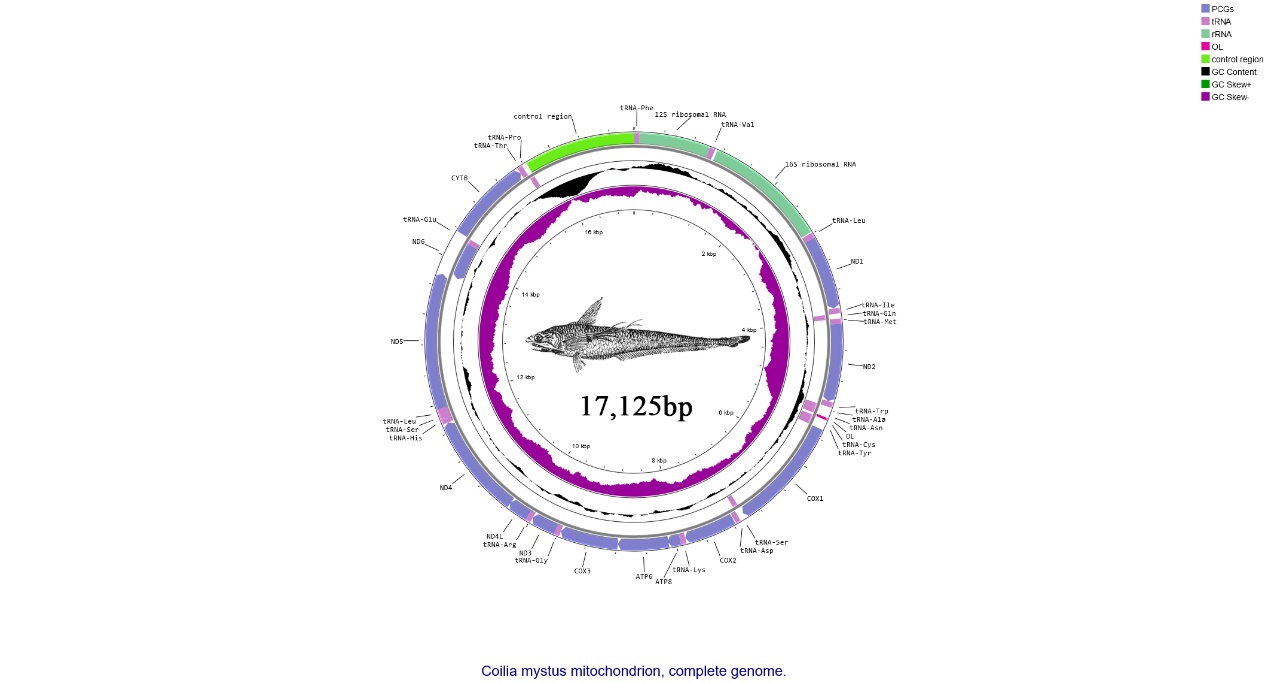

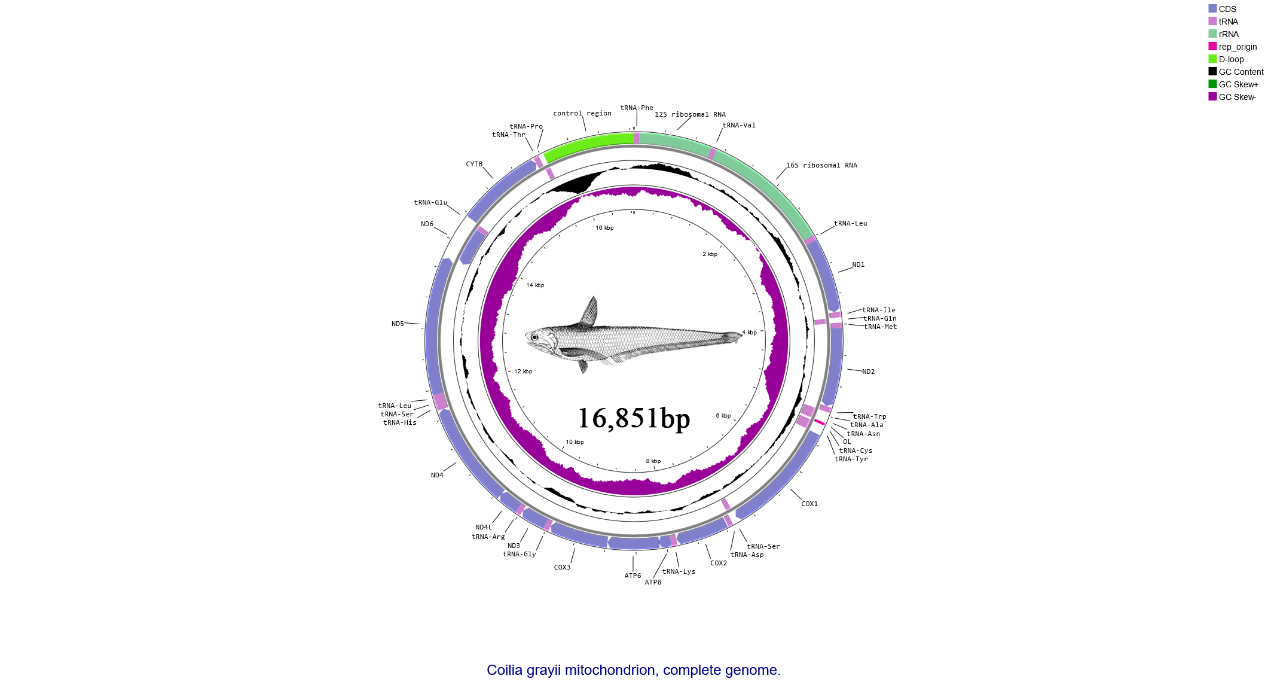

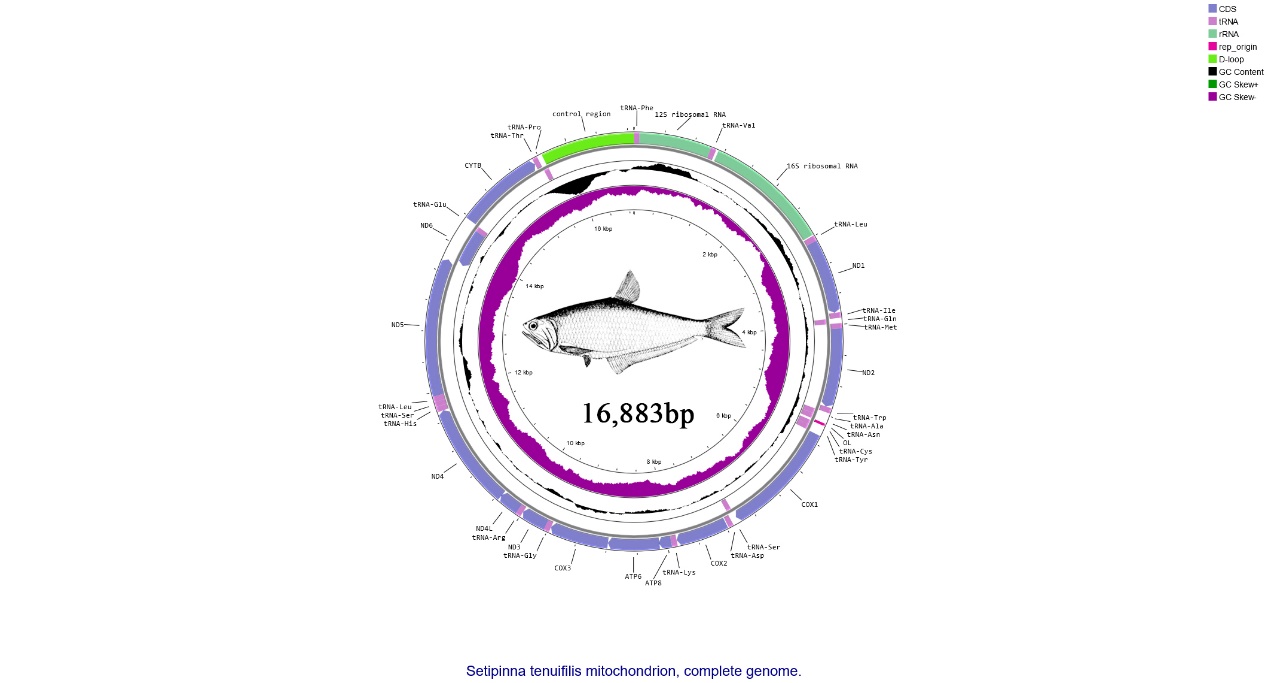

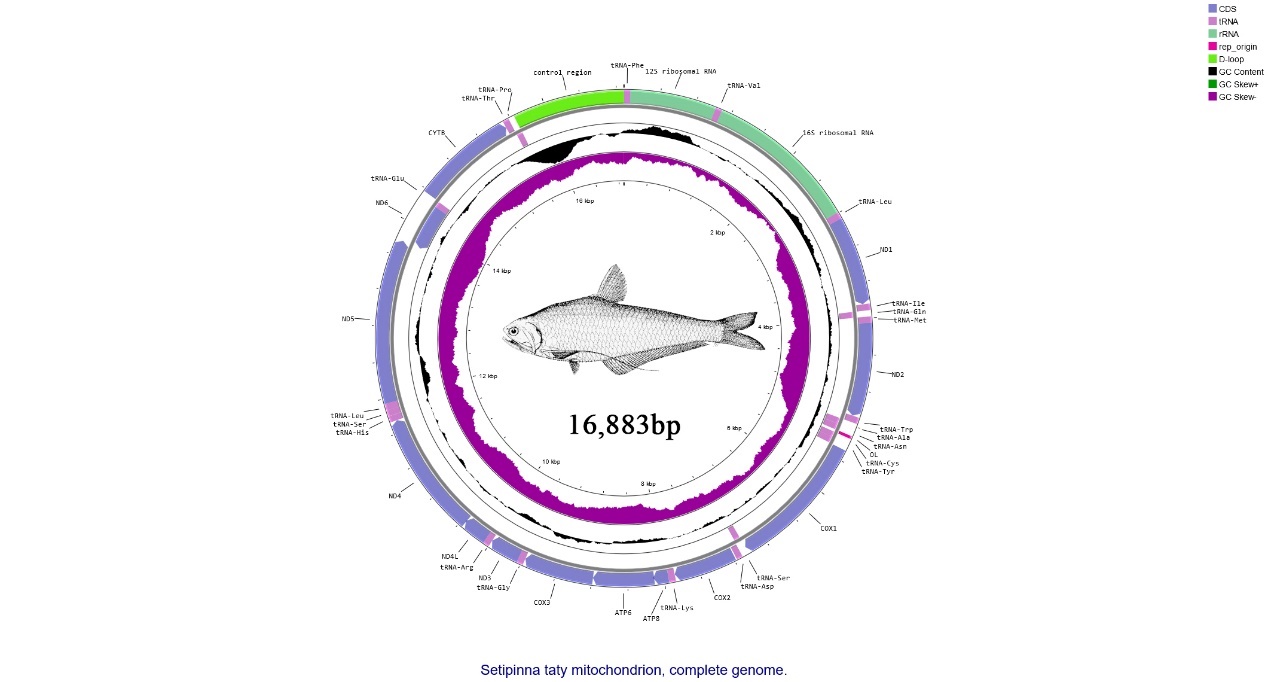

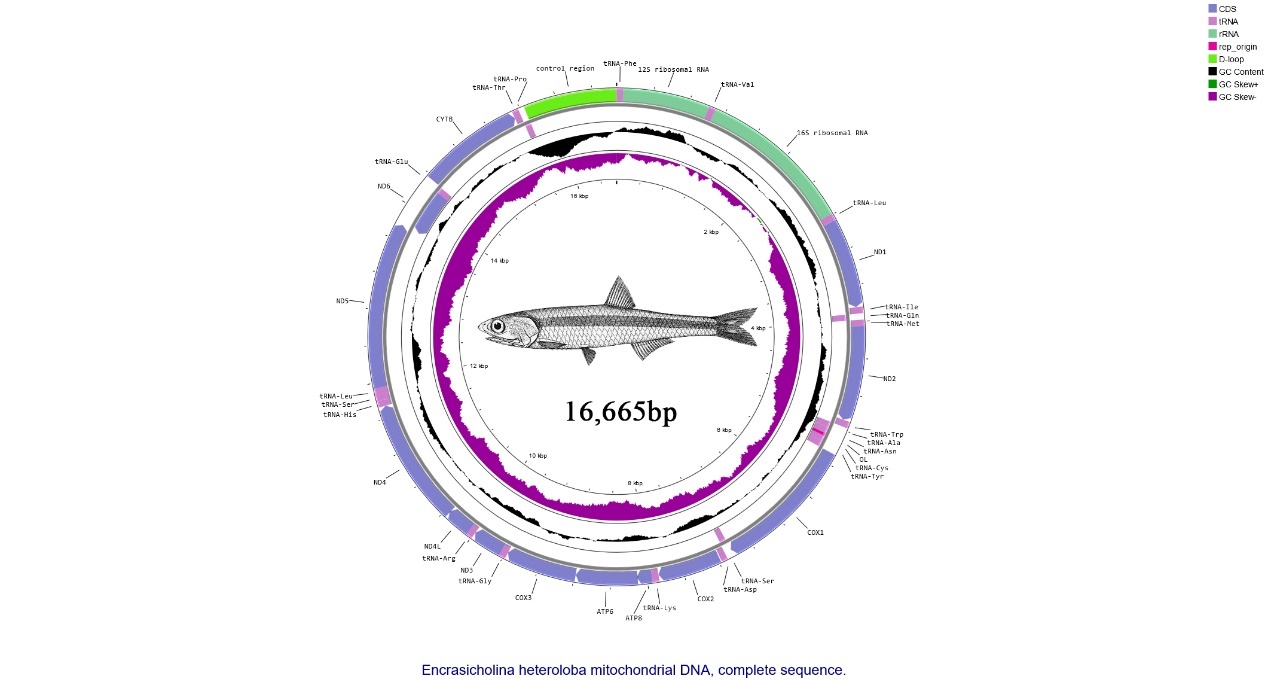

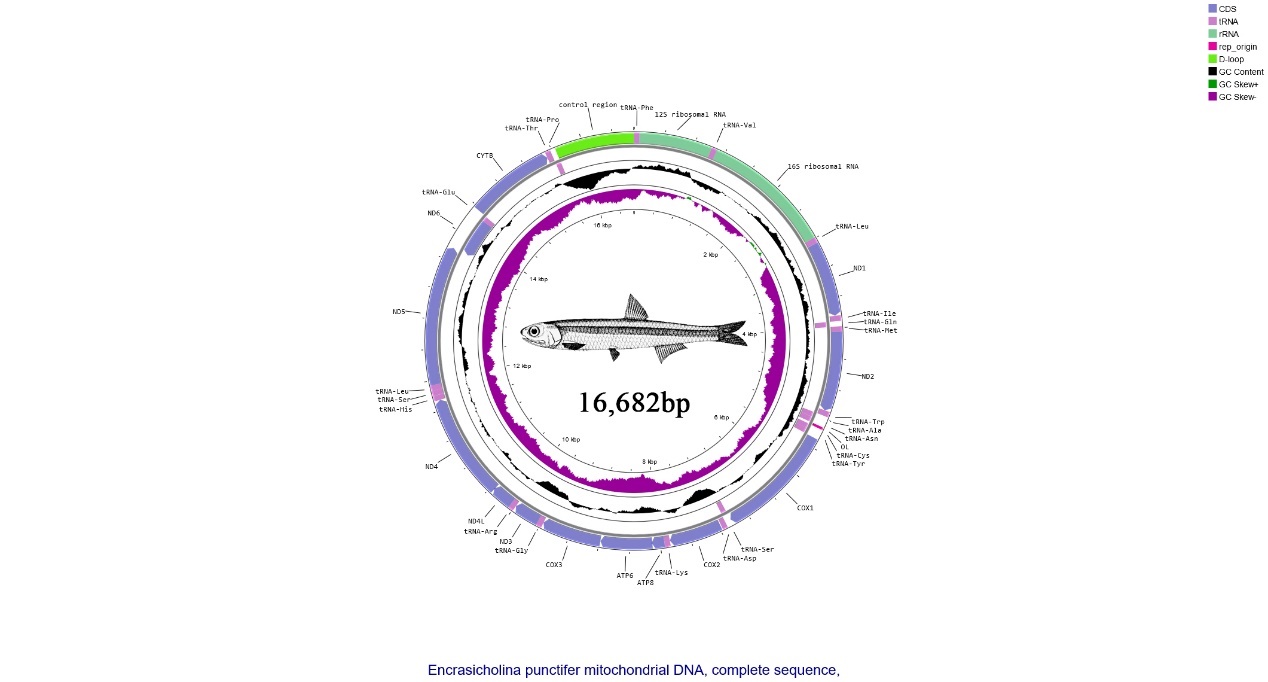

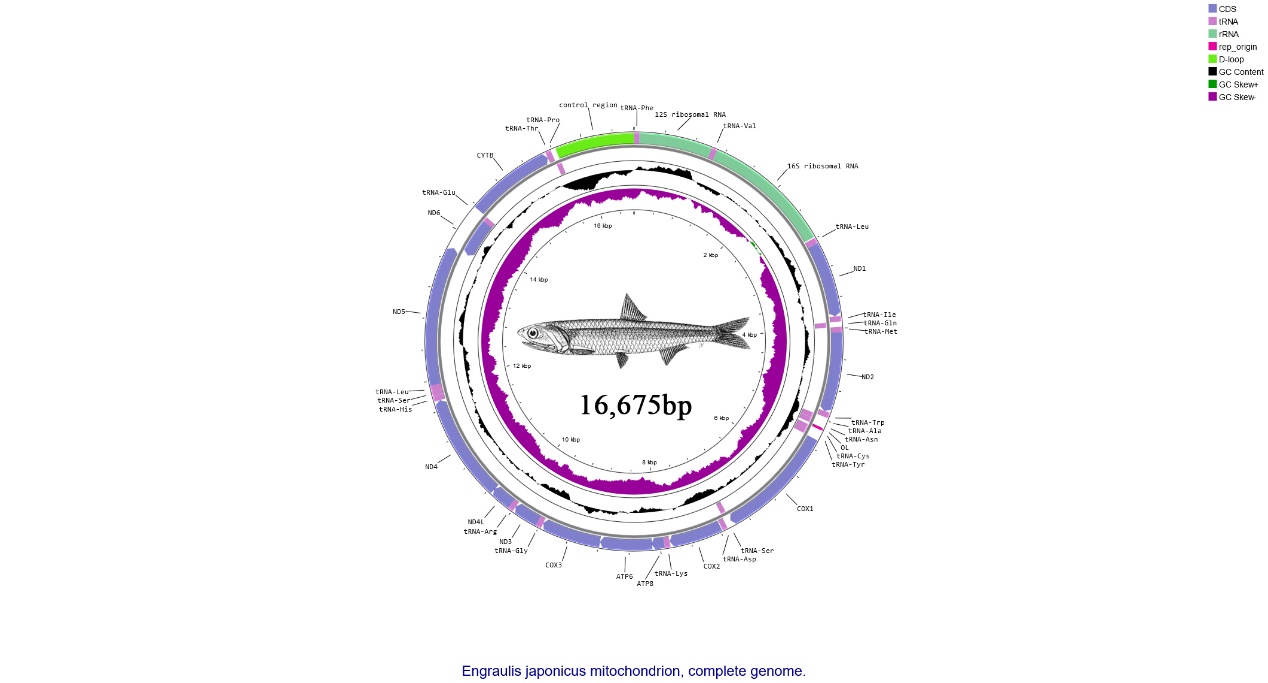

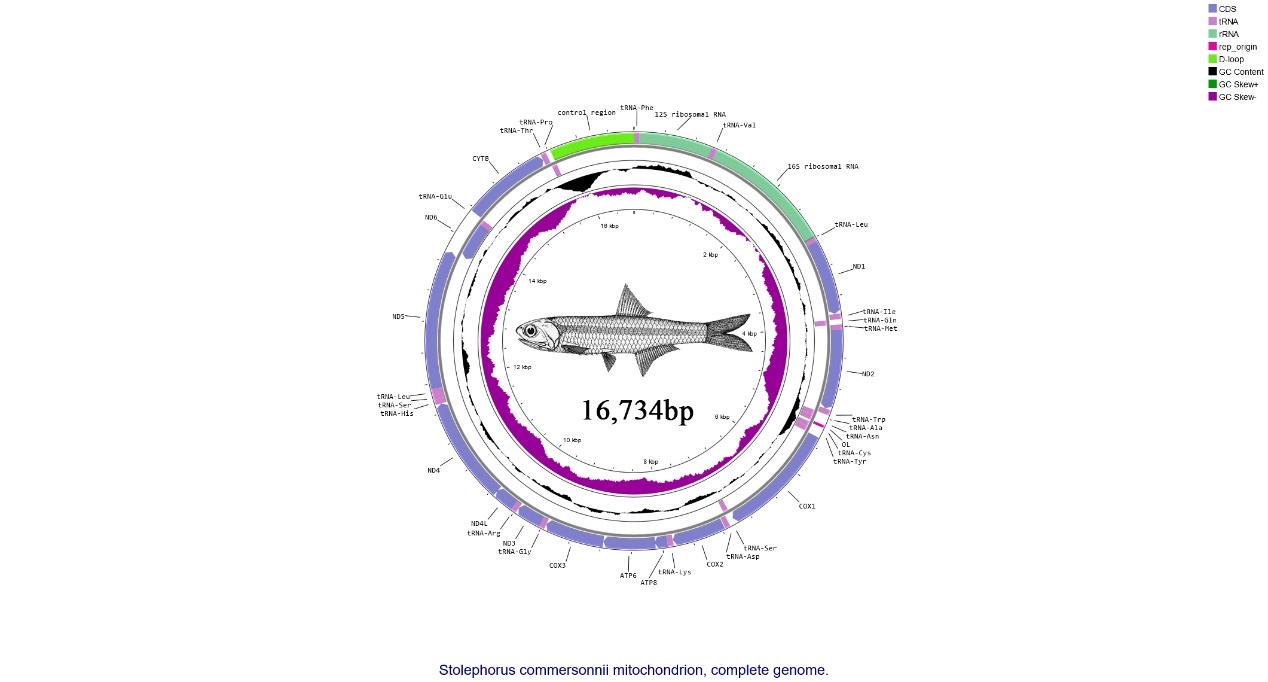

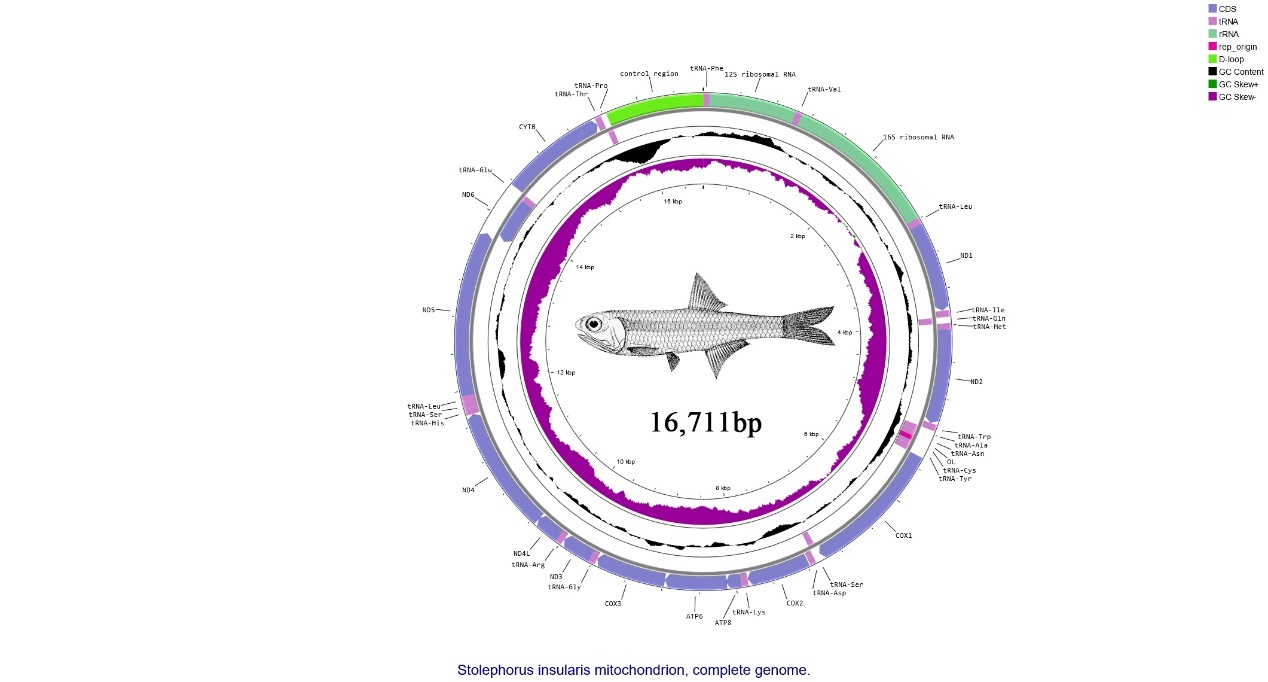

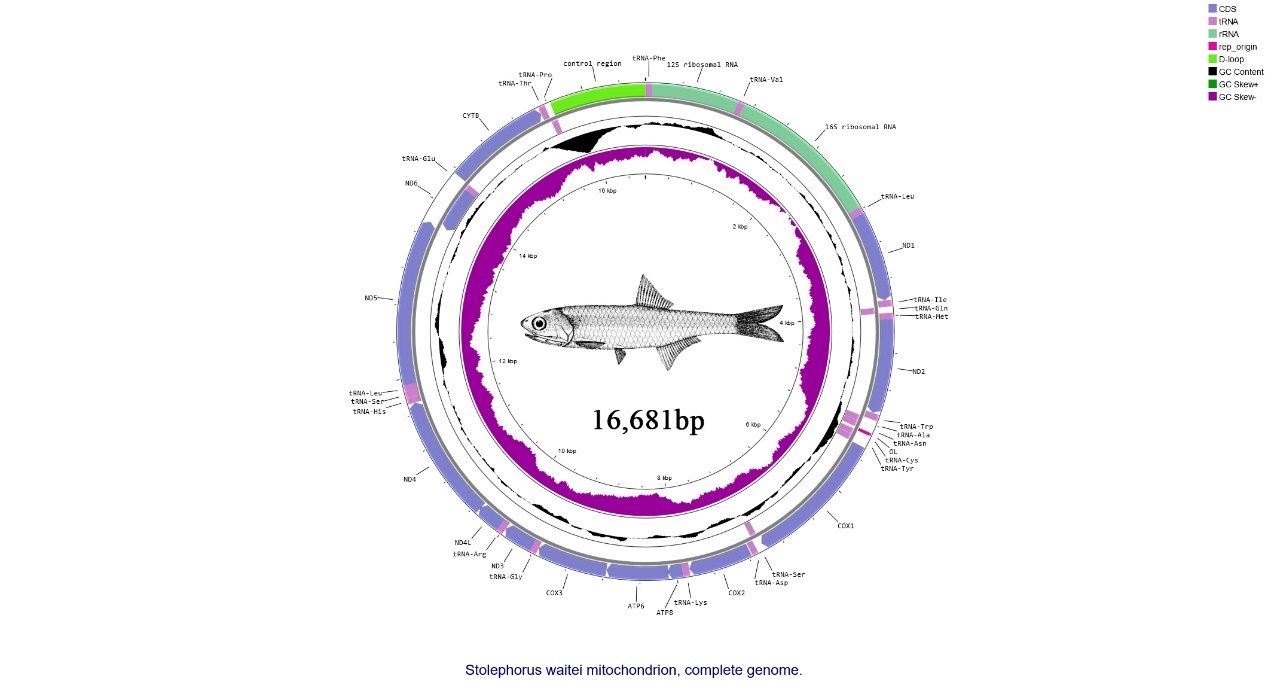
**

**h**

**i**

**j**

**k**

**l**

**m**

**n**

**o**

**p**

**q**

**r**

**Figure S2. Phylogenetic trees constructed using five datasets.** **Red numbers represent posterior probabilities or ML bootstrap, while black numbers represent genetic distances.**

**(a. PCGs-ML, b. PCGs-BI, c. PCGs12-ML, d. PCGs12-BI, e. PCGsRNA-ML, f. PCGsRNA-BI, g. PCGs12RNA-ML, h. PCGs12RNA-BI, i. Mt-ML, j. Mt-BI.)**

**
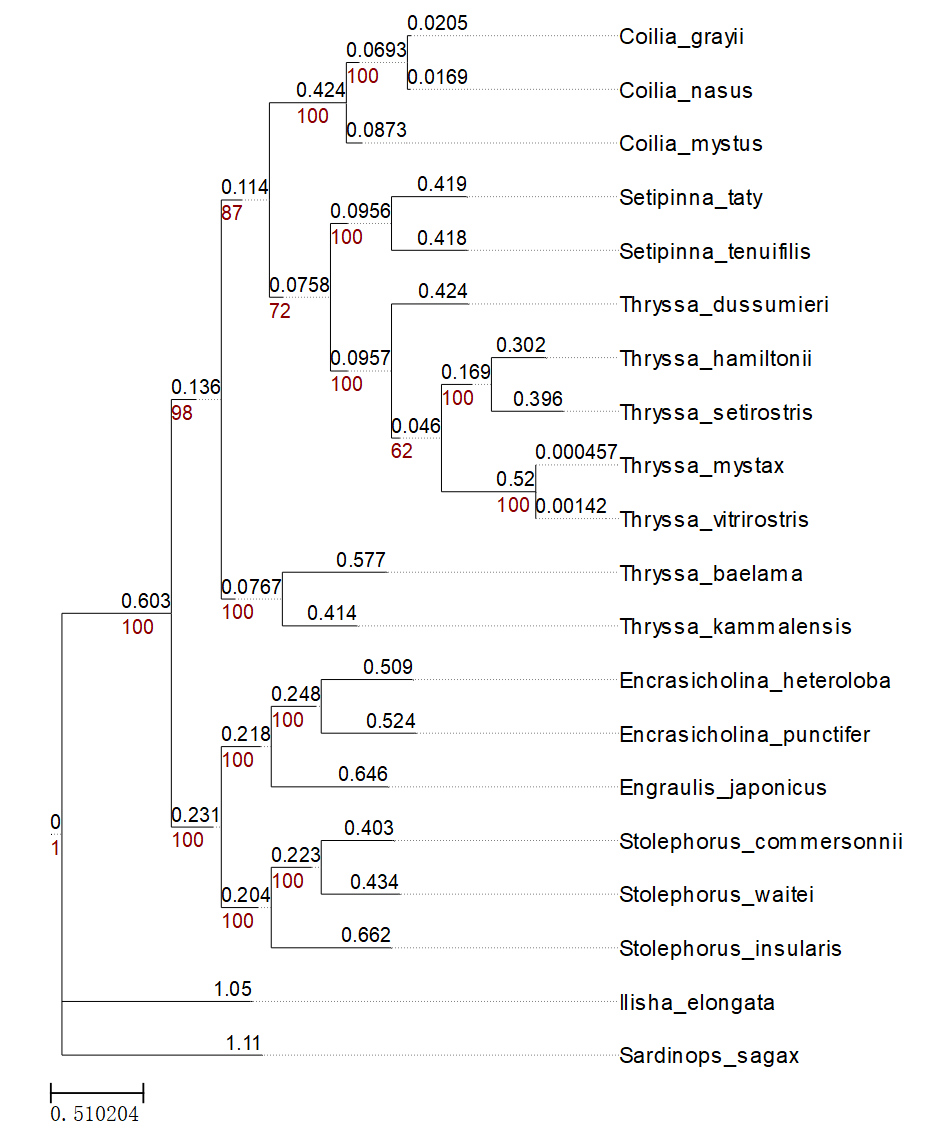
**

**a**

**PCGs-ML**

**
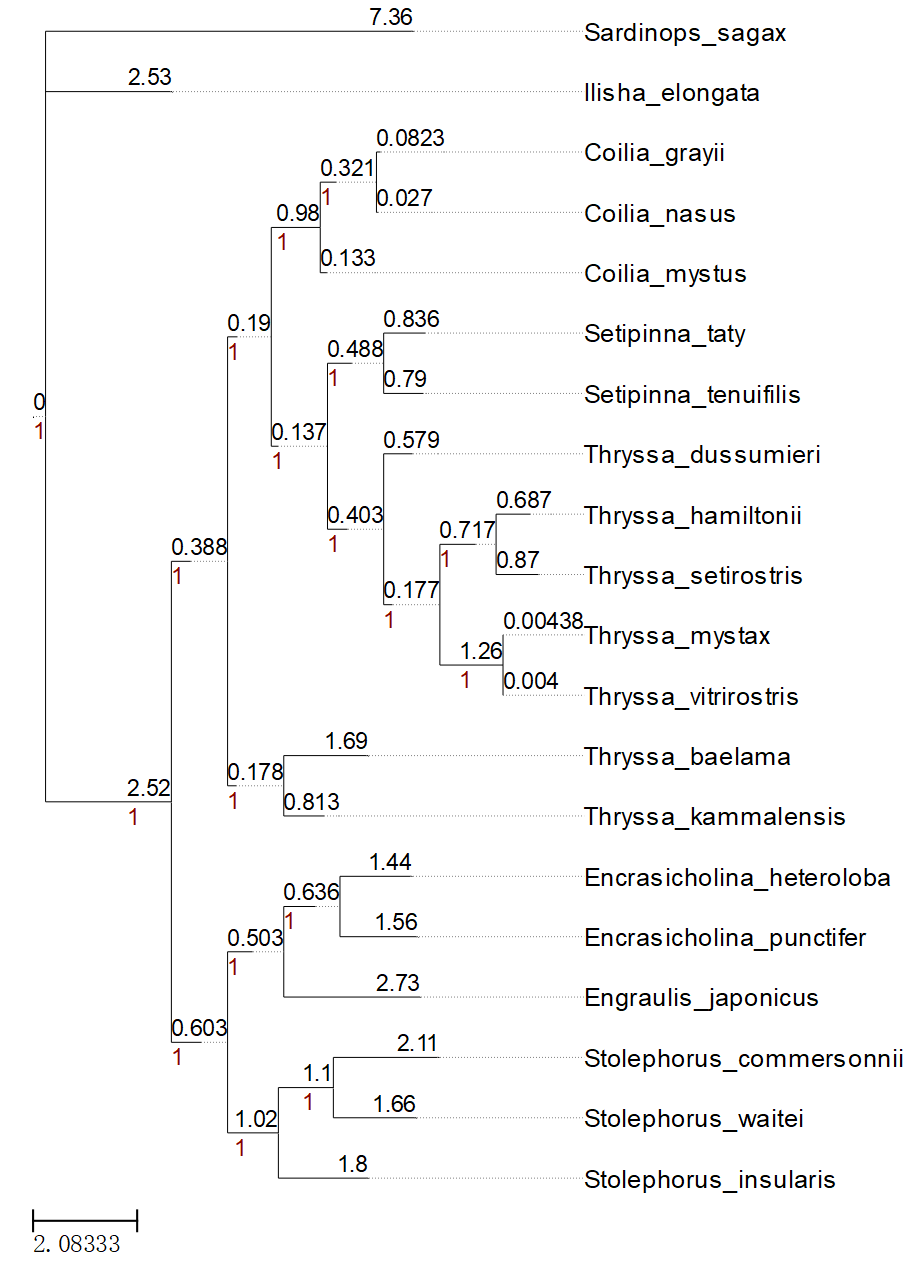
**

**b**

**PCGs-BI**

**
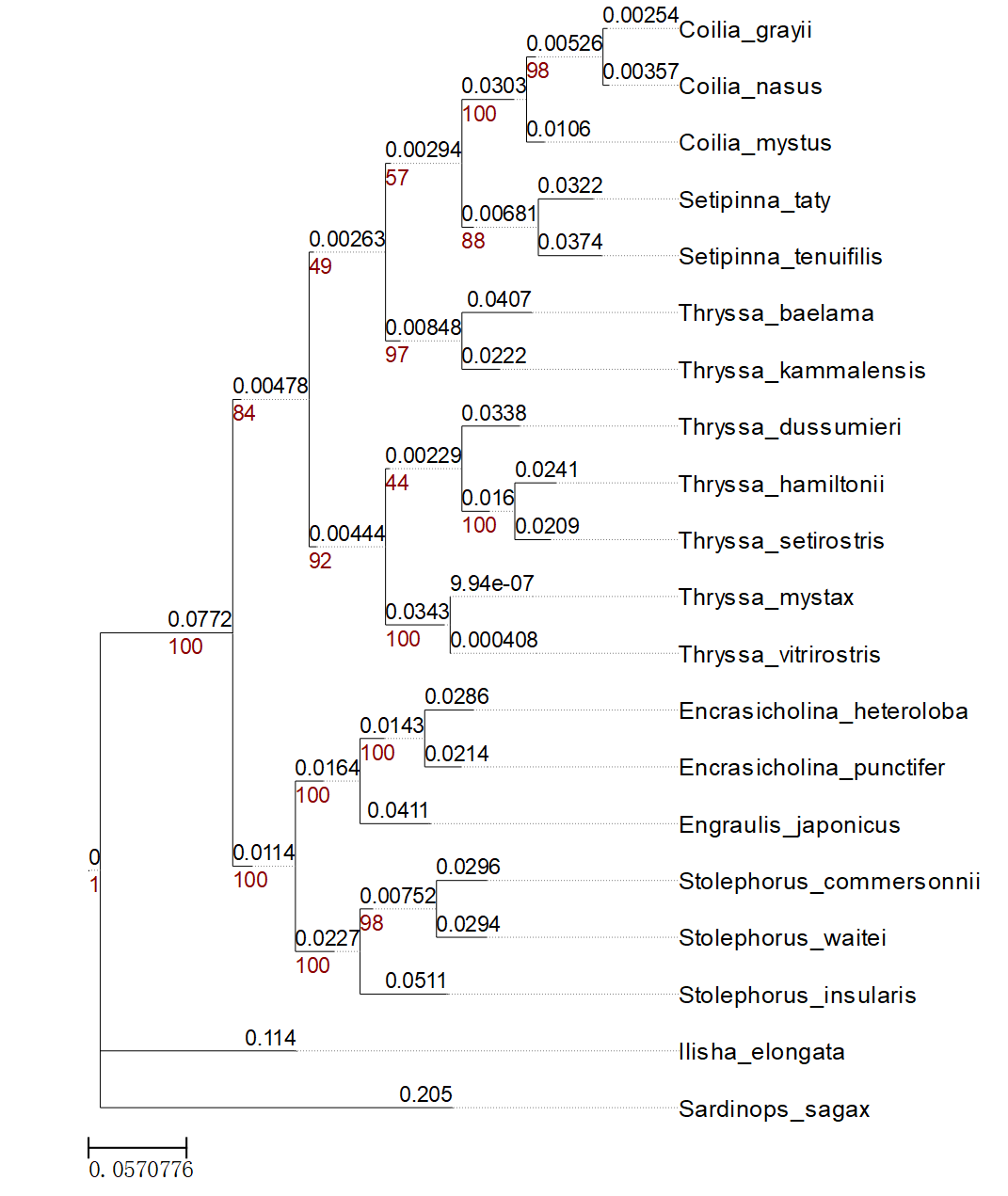
**

**c**

**PCGs12-ML**

**
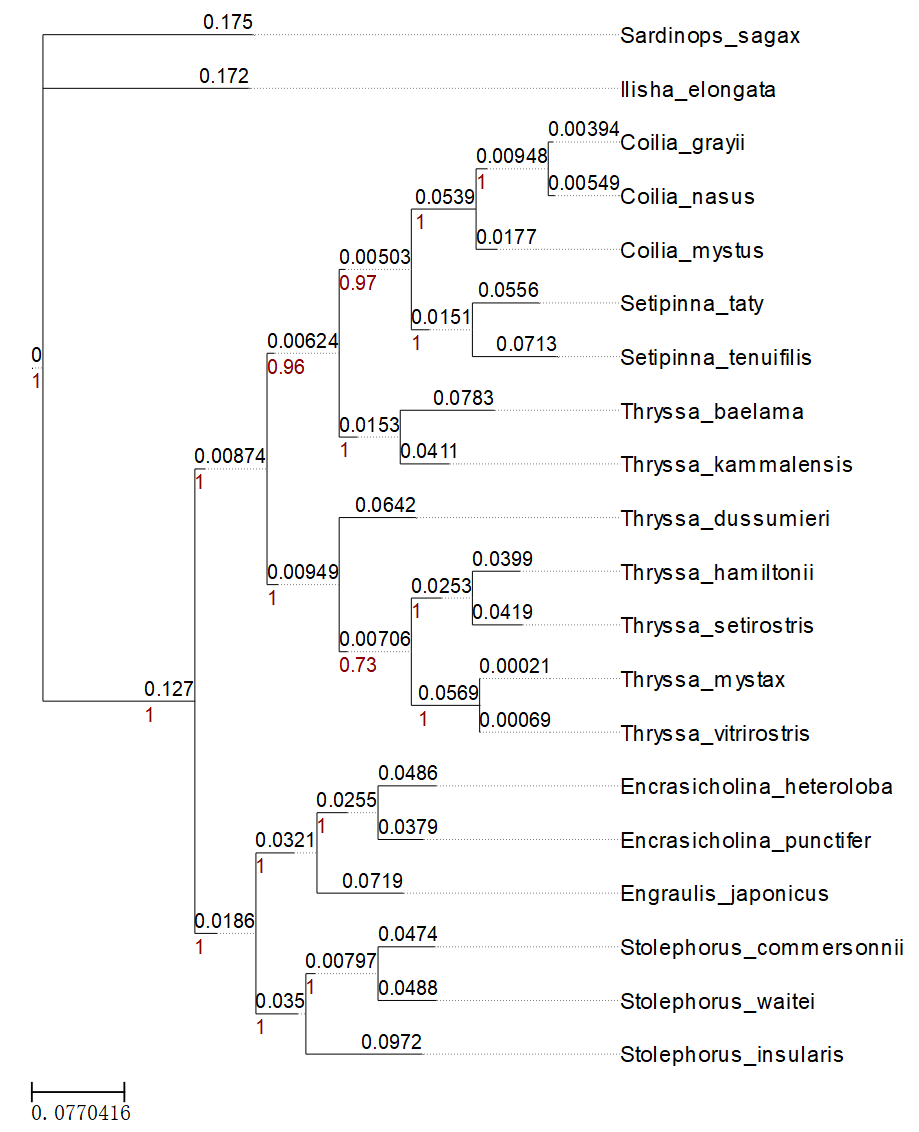
**

**d**

**PCGs12-BI**

**
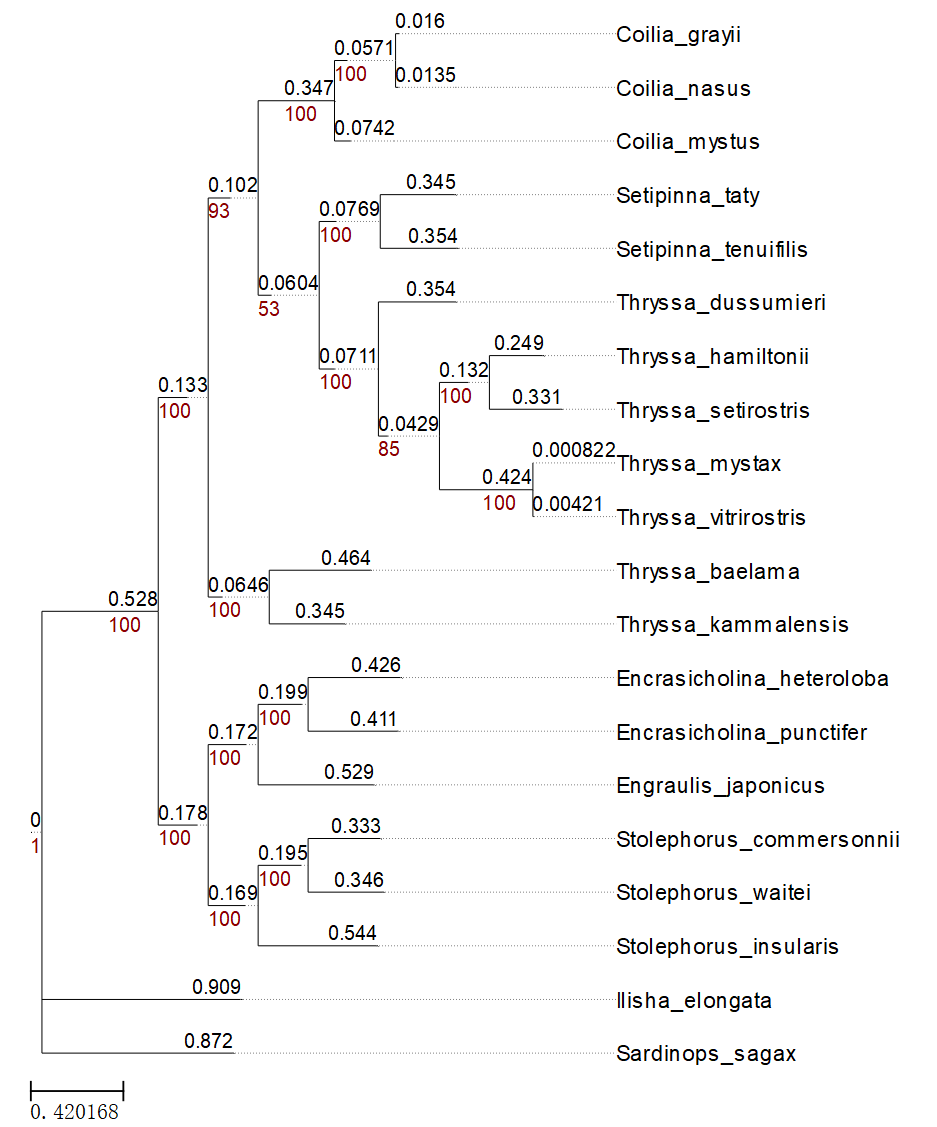
**

**e**

**PCGsRNA-ML**

**
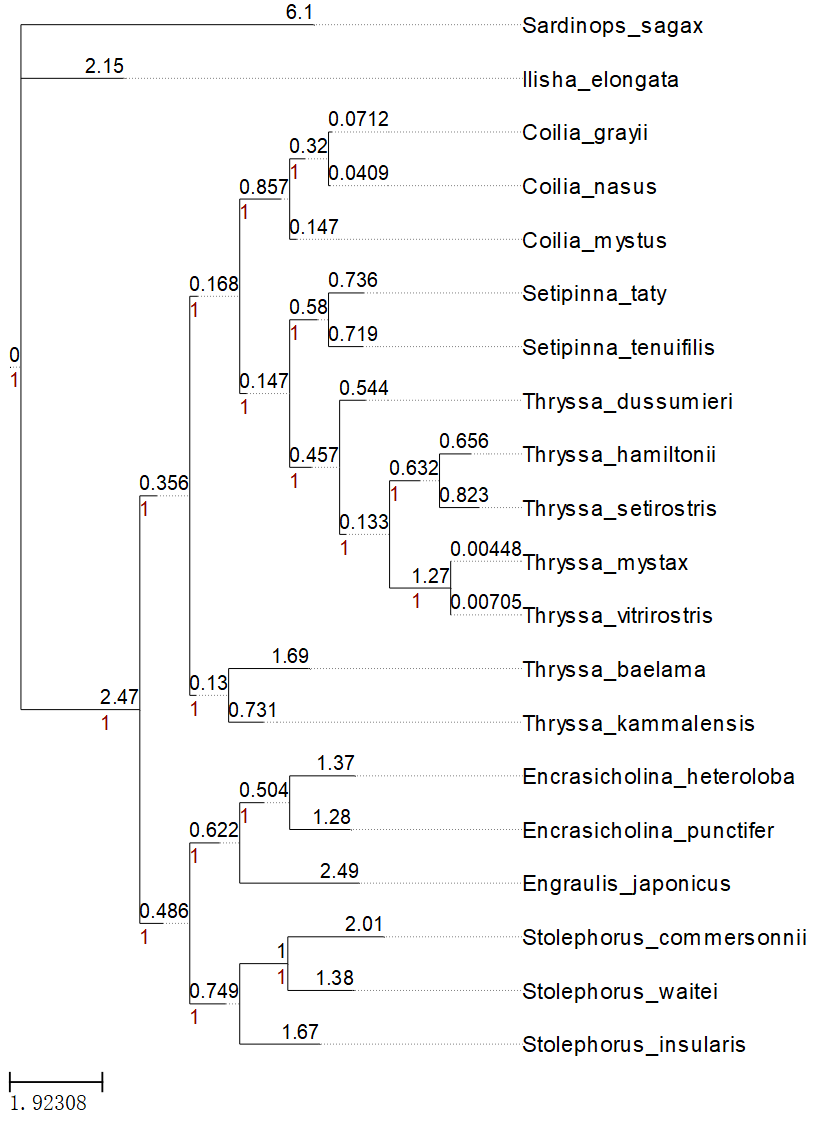
**

**f**

**PCGsRNA-BI**

**
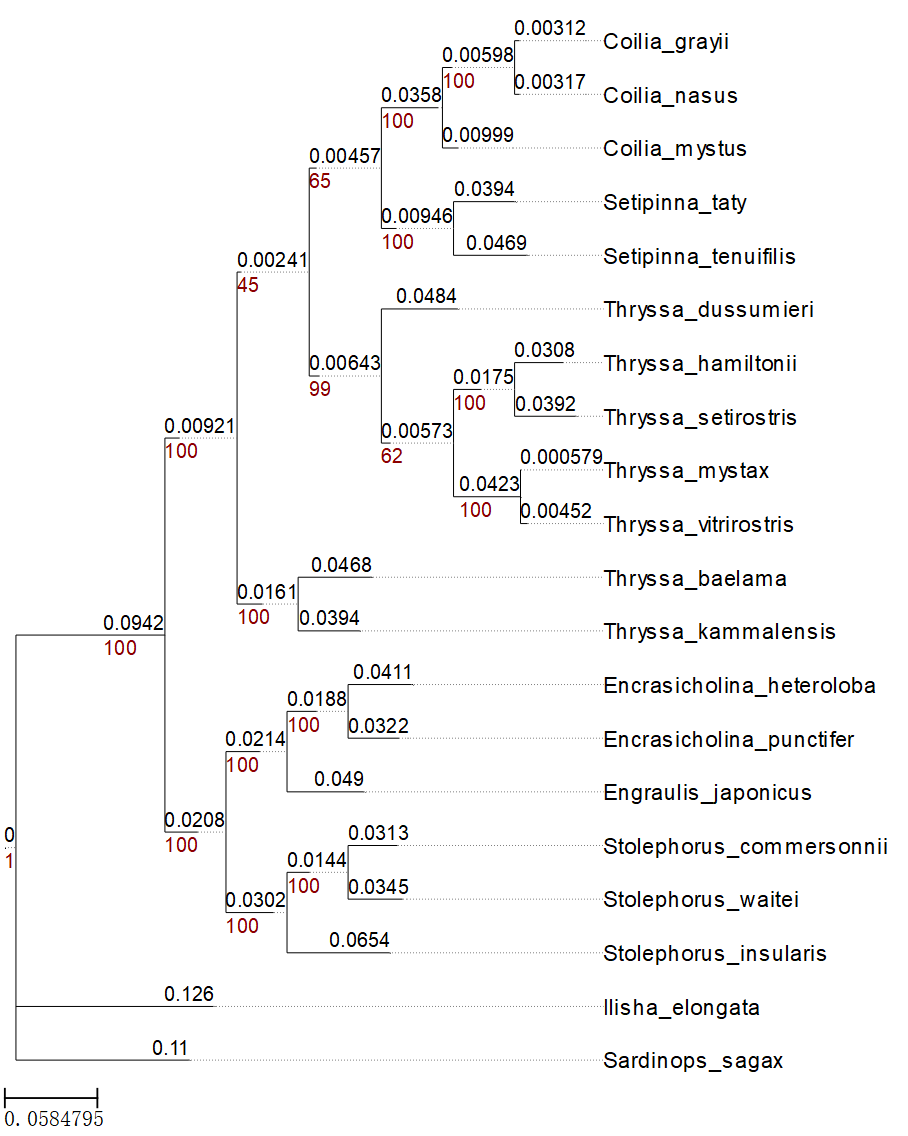
**

**g**

**PCGs12RNA-ML**

**
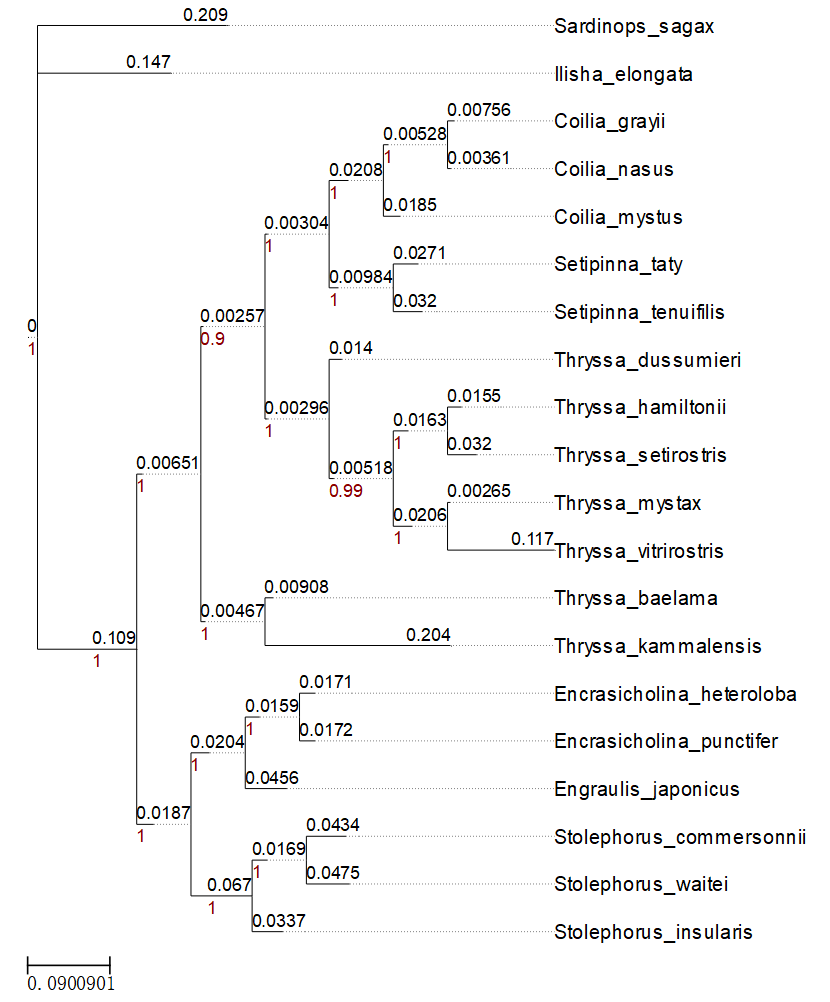
**

**h**

**PCGs12RNA-BI**

**
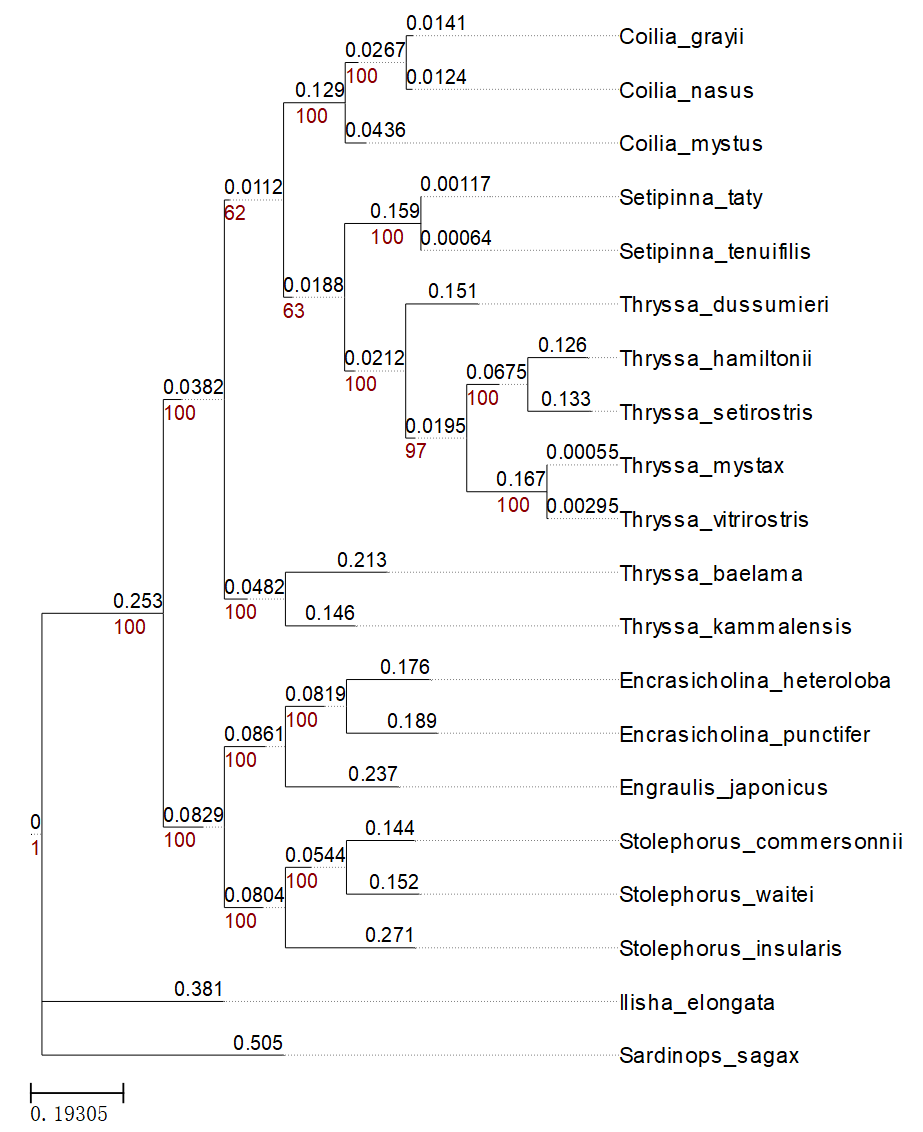
**

**i**

**Mt-ML**

**j**

**
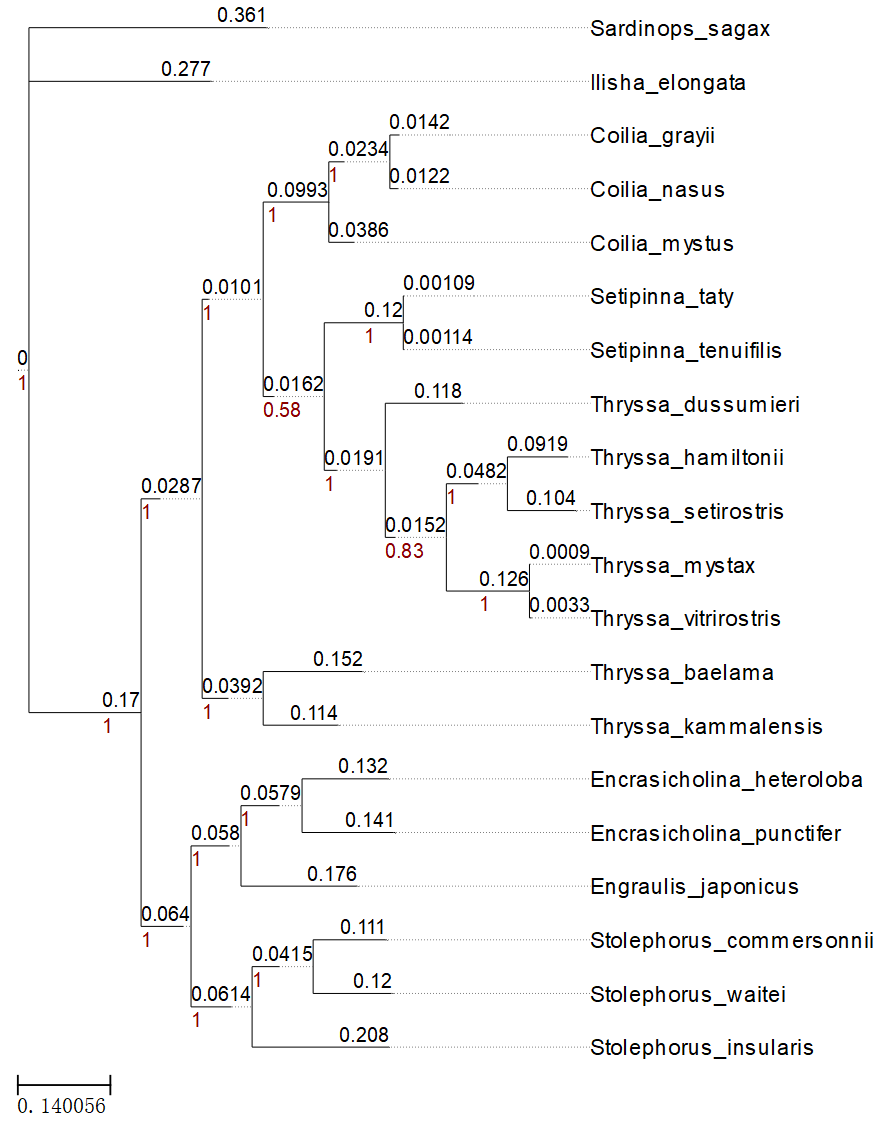
**

**Mt-BI**

**Figure S3.** **Substitution saturation analysis for the four datasets. The higher the R² value and the closer the slope is to 1, the lower the level of saturation.(a. PCGs, b.PCGs12, c. PCGsRNA, d. PCGs12RNA)**

**a**

**
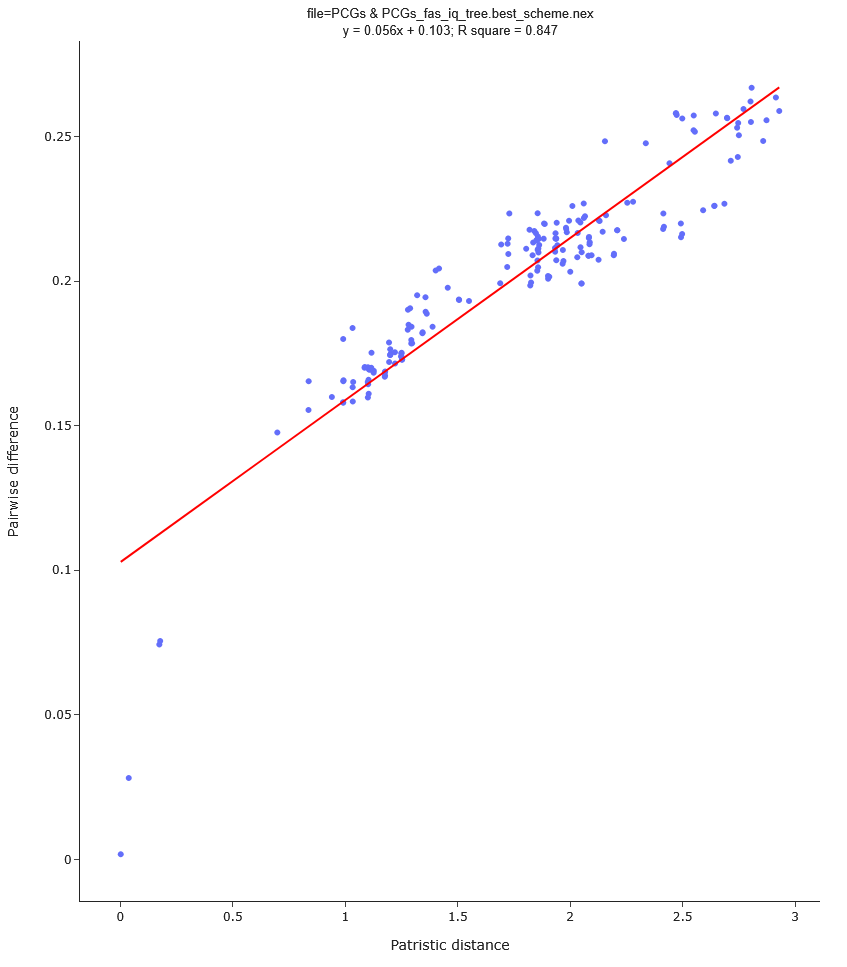
**

**
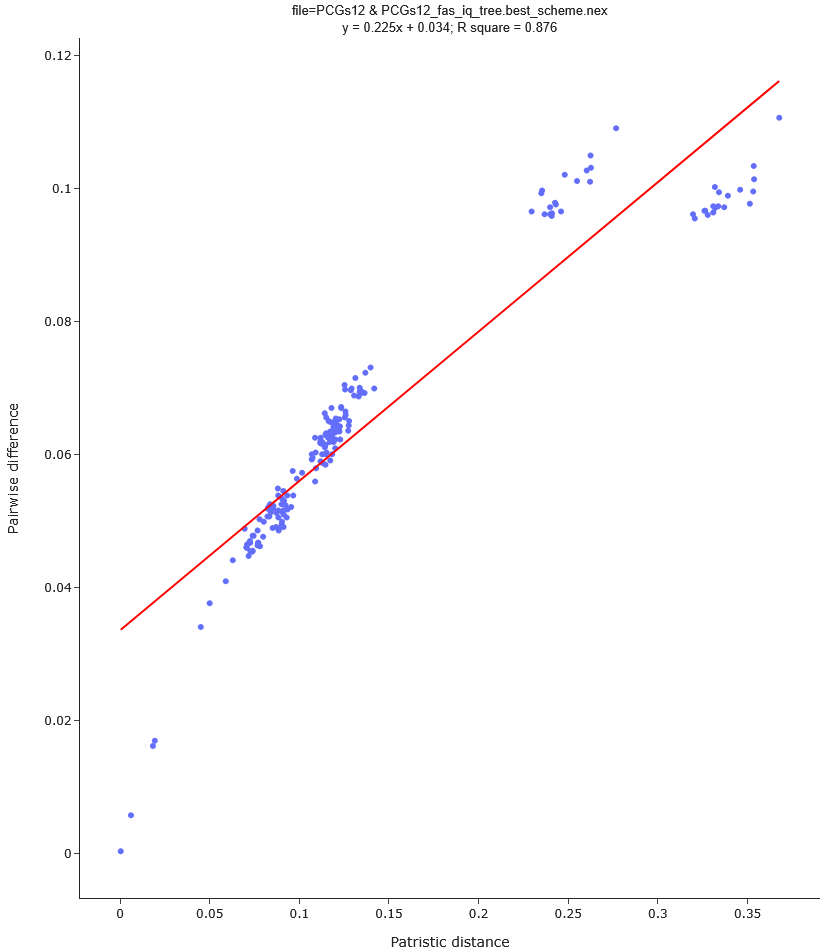
**

**b**

**
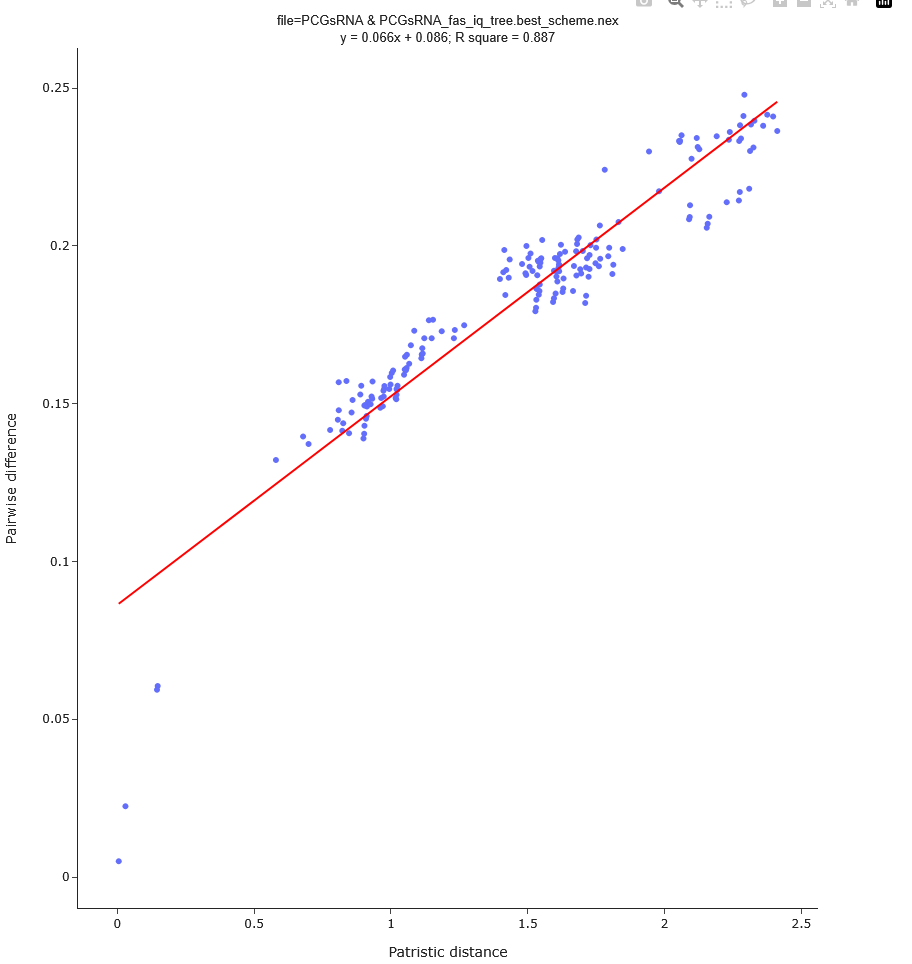
**

**c**

**
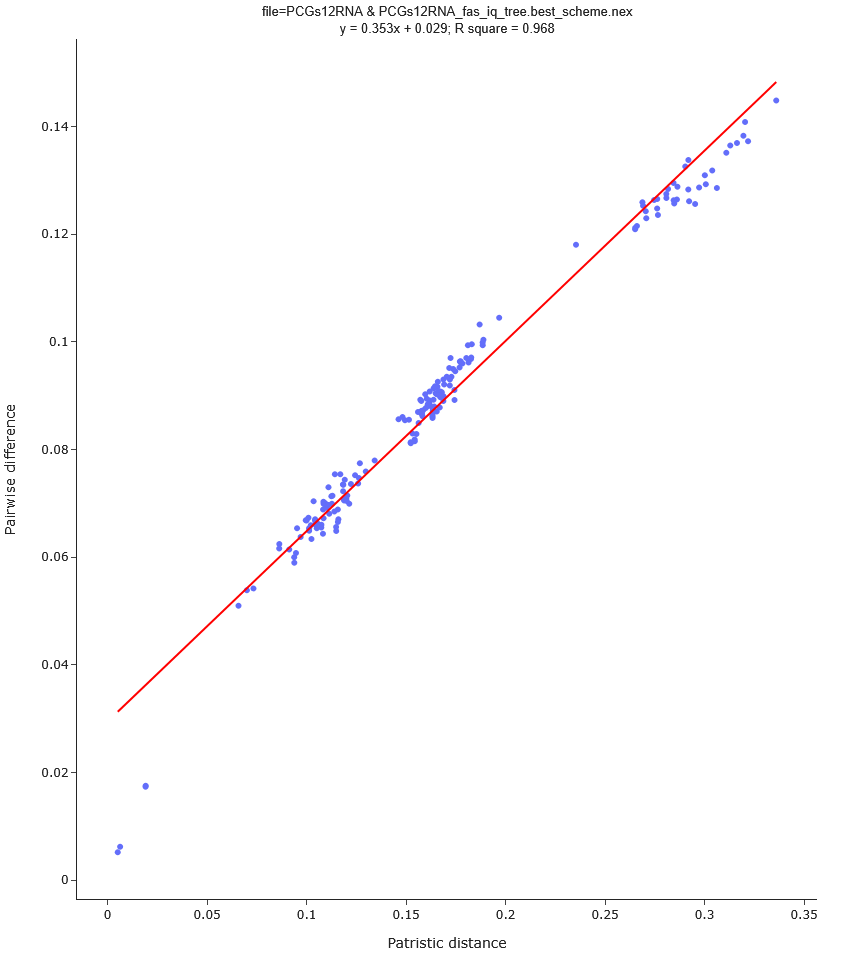
**

**d**
